# Epidemiology and disease burden of patients requiring neurocritical care: a Brazilian multicentre cohort study

**DOI:** 10.1038/s41598-023-44261-w

**Published:** 2023-10-30

**Authors:** Álvaro Réa-Neto, Rafaella Stradiotto Bernardelli, Mirella Cristine de Oliveira, Paula Geraldes David-João, Amanda Christina Kozesinski-Nakatani, Antônio Luís Eiras Falcão, Pedro Martins Pereira Kurtz, Hélio Afonso Ghizoni Teive, Neurocritical Brazil Study group, Fabíola Prior Caltabeloti, Salomon Soriano, Viviane Cordeiro Veiga, Fernando Augusto Bozza, Luana Alves Tannous, Juliano Gasparetto, Fernanda Sampaio Alves, José Arthur Santos Brasil, Glécia Carla Rocha, Jarbas Motta Junior, Bruna Martins Dzivielevski Câmara, Livia Rodrigues Figueiredo, Janaína Oliveira, William Nascimento Vianna, Diogo Roberto Lorenzo Iglesias, Rafael Alexandre de Oliveira Deucher, Gloria Martins, Marcel Resende Lopes, Frederico Bruzzi de Carvalho, Jorge Luiz da Rocha Paranhos, Ulysses Vasconcellos de Andrade e Silva, Marco Oliveira Py, Fernanda Baeumle Reese, Marcos Freitas Knibel, Gustavo Cartaxo Patriota, Suzana Margareth Ajeje Lobo, Mario Roberto Rezende Guimarães Junior, Luciana de Oliveira Neves, Antônio Aurélio Fagundes, Ary Serpa Neto, Walter Carlos Girardelli Baptista, Cintia Magalhães Carvalho Grion, Péricles Almeida Delfino Duarte, Bruno Branco, Luísa da Silva André Salgado, Nívea Melo de Souza Costa, Danilo Bastos Pompermayer, Anna Flavia Kaled, Rafael Brum, Alessandro Rocha Milan de Souza, Jackson Erasmo Fuck, Claudio Piras

**Affiliations:** 1 Center for Studies and Research in Intensive Care Medicine (CEPETI), Curitiba, Brazil; 2 Internal Medicine Department, Hospital de Clínicas, Federal University of Paraná, Curitiba, Paraná Brazil; 3 Neurological Institute of Curitiba Hospital, Curitiba, Paraná Brazil; 4 School of Medicine and Life Sciences, Pontifical Catholic University of Paraná, Curitiba, Paraná Brazil; 5 Complexo Hospitalar do Trabalhador (CHT), Curitiba, Paraná Brazil; 6 Department of Critical Patients, Hospital Municipal Dr Moysés Deutsch, São Paulo, São Paulo Brazil; 7 Hospital Santa Casa de Curitiba, Curitiba, Paraná Brazil; 8 Medical School, University of Campinas (UNICAMP), Campinas, São Paulo Brazil; 9 Head of the Intensive Care Unit, Hospital de Clínicas de Campinas, Campinas, São Paulo Brazil; 10 D’Or Institute of Research and Education, Rio de Janeiro, Rio de Janeiro Brazil; 11 Hospital Copa Star, Rio de Janeiro, Rio de Janeiro Brazil; 12 Instituto Estadual do Cérebro Paulo Niemeyer, Rio de Janeiro, Rio de Janeiro Brazil; 13 Neurology Service, Movement Disorders Unit, Internal Medicine Department, Hospital de Clínicas, Federal University of Paraná, Curitiba, Paraná Brazil; 14 Postgraduate Program in Internal Medicine, Neurological Diseases Group, Federal University of Paraná, Curitiba, Paraná Brazil; 15 Hospital das Clínicas da Faculdade de Medicina da Universidade de São Paulo, São Paulo, São Paulo Brazil; 16 Hospital Beneficência Portuguesa de São Paulo, São Paulo, São Paulo Brazil; 17 Hospital Copa D’Or, Rio de Janeiro, Rio de Janeiro Brazil; 18 Hospital Barra D’Or, Rio de Janeiro, Rio de Janeiro Brazil; 19 Hospital Caxias D’Or, Rio de Janeiro, Rio de Janeiro Brazil; 20 Hospital Quinta D’Or, Rio de Janeiro, Rio de Janeiro Brazil; 21 Hospital Universitário Cajuru, Curitiba, Paraná Brazil; 22 Hospital da Bahia, Salvador, Bahia, Brazil; 23 Hospital de Base do Distrito Federal, Brasília, Distrito Federal Brazil; 24 Hospital Marcelino Champagnat, Curitiba, Paraná Brazil; 25 Hospital Municipal de Cuiabá, Cuiabá, Mato Grosso Brazil; 26 Hospital Vita Batel, Curitiba, Paraná Brazil; 27 Santa Casa de Misericórdia de Passos, Passos, Minas Gerais Brazil; 28 Fundação Hospitalar do Estado de Minas Gerais, Belo Horizonte, Minas Gerais Brazil; 29 Santa Casa São João del Rei, São João del Rei, Minas Gerais Brazil; 30 Hospital de Amor, Barretos, São Paulo Brazil; 31 Hospital São Lucas Copacabana, Rio de Janeiro, Rio de Janeiro Brazil; 32 Hospital Estadual de Emergência e Trauma Senador Humberto Lucena, João Pessoa, Paraíba Brazil; 33 Hospital de Base de Rio Preto, São José do Rio Preto, São Paulo Brazil; 34 Hospital São Carlos, Fortaleza, Ceará Brazil; 35 Hospital HOME, Brasília, Distrito Federal Brazil; 36 Hospital Israelita Albert Einstein, São Paulo, São Paulo Brazil; 37 Hospital Novo Atibaia, Atibaia, São Paulo Brazil; 38 Hospital Universitário da Universidade Estadual de Londrina, Londrina, Paraná Brazil; 39 Hospital Universitário do Oeste do Paraná, Cascavel, Paraná Brazil; 40 Hospital das Nações, Curitiba, Paraná Brazil; 41 Hospital de Força Aérea do Galeão, Rio de Janeiro, Rio de Janeiro Brazil; 42 Hospital de Clínicas Antônio Paulino-Pronil, Niteroi, Rio de Janeiro Brazil; 43 Hospital do Câncer UOPECCAN, Umuarama, Paraná Brazil; 44 Vitoria Apart Hospital, Serra, Espírito Santo Brazil

**Keywords:** Epidemiology, Neurological disorders, Neurological disorders

## Abstract

Acute neurological emergencies are highly prevalent in intensive care units (ICUs) and impose a substantial burden on patients. This study aims to describe the epidemiology of patients requiring neurocritical care in Brazil, and their differences based on primary acute neurological diagnoses and to identify predictors of mortality and unfavourable outcomes, along with the disease burden of each condition at intensive care unit admission. This prospective cohort study included patients requiring neurocritical care admitted to 36 ICUs in four Brazilian regions who were followed for 30 days or until ICU discharge (Aug-Sep in 2018, 1 month). Of 4245 patients admitted to the participating ICUs, 1194 (28.1%) were patients with acute neurological disorders requiring neurocritical care and were included. Patients requiring neurocritical care had a mean mortality rate 1.7 times higher than ICU patients not requiring neurocritical care (17.21% versus 10.1%, respectively). Older age, emergency admission, higher number of potential secondary injuries, and worse APACHE II, SAPS III, SOFA, and Glasgow coma scale scores on ICU admission are independent predictors of mortality and poor outcome among patients with acute neurological diagnoses. The estimated total DALYs were 4482.94 in the overall cohort, and the diagnosis with the highest DALYs was traumatic brain injury (1634.42). Clinical, epidemiological, treatment, and ICU outcome characteristics vary according to the primary neurologic diagnosis. Advanced age, a lower GCS score and a higher number of potential secondary injuries are independent predictors of mortality and unfavourable outcomes in patients requiring neurocritical care. The findings of this study are essential to guide education policies, prevention, and treatment of severe acute neurocritical diseases.

## Introduction

A proper understanding of the impact that a disease has on a population is a crucial step for the implementation of preventive, therapeutic, and rehabilitative measures^1^. Neurological disorders have an increasingly high prevalence and impose a substantial burden on patients, families, and society in general^2^. Patients who are critically ill with neurological or neurosurgical diseases require neurocritical care to treat the primary insult to the nervous system and prevent or ameliorate secondary neurological and nonneurological injuries. Patients with diverse acute neurological disorders constitute a considerable proportion of all admissions to intensive care units (ICUs) worldwide. In Brazil, approximately 10% of ICU admissions are due to neurological causes, according to monitoring data from Brazilian UTIS from 2010 to 2023^3^. They have high morbidity and mortality and consume substantial health care resources, and those who survive progress with relevant and persistent disabilities^4–6^. However, the relative distribution of patients requiring neurocritical care and their burden on health care services remain unclear.

Identifying the proportions, severities, and outcomes of these patients can provide goals to optimize the use of available resources, support protocols and processes, and improve medical education^7,8^. The Neurocritical Brazil Study aimed to better describe the epidemiology of patients requiring neurocritical care in Brazil and their differences based on primary acute neurological disorders and to identify predictors of mortality and poor outcomes, along with an estimate of the disease burden of each acute neurological disorder group identified at ICU admission.

## Methods

We conducted a national prospective cohort study including all patients with primary diagnoses of acute neurological conditions admitted to 36 ICUs over 30 consecutive days. After admission, patients were followed for 30 days or until ICU discharge.

The study was approved by the local ethics committee of the Neurological Institute of Curitiba (ethics committee of the coordinating centre) on April 20, 2017 (approval number 2.024.132) and by the local ethics committees at each participating centres. The need for informed consent was waived in all centres, given the noninterventional design of the study and the fact that the data were collected from clinical records and without contact with the participants. All research procedures were conducted in accordance with the ethical standards of the committees on human experimentation of each participating institution and the Declaration of Helsinki (7th revision, 2013). The study results are reported in accordance with the Strengthening the Reporting of Observational Studies in Epidemiology (STROBE) guidelines.

We invited all 300 ICU members of the ICU network identified in the ICU network of the Brazilian Association of Intensive Care Medicine (AMIBnet) to participate in the study. To participate in the study, each ICU should have an active database of patients, a coordinator willing to follow up on all patients requiring neurocritical care for up to 30 consecutive days, and a team available for data collection. Each ICU should also obtain timely study protocol approval by the institution’s ethics committee. In all, 80 ICUs agreed to participate and 36 were included and recruited patients for the study. These ICUs were in four of the five most populous Brazilian regions.

The participating ICUs were distributed across various Brazilian regions. The Supplementary Material S1 presents a complete list of the centres of all participating ICUs and their corresponding investigators, the distribution of participating enrolling centres across Brazil (Supplementary Fig. S1) and the percentage of contribution from each participating centre to the sample (Supplementary Fig. S2).

The study data were collected in 2018 between August 1–30 (31 ICUs) and September 1–30 (5 ICUs). All patients admitted to the participating ICUs during the 30 days of the study were screened, and those with an acute neurological disorder that was the primary cause of ICU admission were consecutively included in the study and followed up for only 30 days or until ICU discharge. The patients were considered eligible for inclusion if they were older than 18 years and were admitted to a participating ICU during the study period.

The patients were subdivided into ten groups according to their primary acute neurological diagnosis on ICU admission (hereafter, diagnostic groups): ischaemic stroke, intracerebral haemorrhage, subarachnoid haemorrhage, encephalopathy, seizures, traumatic brain injury, spinal cord injury, central nervous system infection, neuromuscular disease, and postoperative care of elective neurosurgery. Patients admitted to the participating ICUs with acute neurological diagnoses different than those listed above were excluded from the study. The group of encephalopathies is made up of several entities that affect the entire brain and alter mental function in a diffuse way, such as, septic encephalopathy, brain structural damage (hydrocephalus, tumour, etc.), metabolic encephalopathy, hypoxic-ischaemic encephalopathy, drug-induced encephalopathy, and other aetiologies.

The Center for Study and Research in Intensive Care Medicine (CEPETI) developed a dedicated electronic case report form to capture the study data. We collected the following variables related to the patients and ICU outcomes: age, sex, comorbidities, type of transportation to the hospital, kind of health care coverage (public, private, or complementary), location before transfer to the ICU, worst clinical scores on severity scales (Glasgow Coma Scale [GCS], Acute Physiology and Chronic Health Evaluation [APACHE] II, Simplified Acute Physiology Score [SAPS] III, Sequential Organ Failure Assessment [SOFA]), potential secondary injuries (the “Hs,” namely, hypotension, hypoxia, hyperthermia, hypercapnia, hypocapnia, hypoglycaemia, hyponatremia, hypothermia, intracranial hypertension, and clinical evidence of herniation—the parameters adopted for the definition of each of the Hs, as well as their cut-off points, are described in Table 1) in the first 24 h in the ICU, the results of neurological imaging tests performed on the first day of ICU admission, complications and procedures performed in the ICU, and length of ICU stay during the 30-day study period. We also collected mortality information and calculated the modified Rankin Scale (mRS) during the 30-day ICU observation period, and both were defined as the study outcomes. Patients with mRS scores of 4, 5, or 6 were considered to have an unfavourable outcome, while those with mRS scores of 0, 1, 2, or 3 were considered to have a favourable outcome.

**Table 1:** Baseline characteristics and procedures, complications, and outcomes during ICU stay among patients requiring neurocritical care.

Characteristics	n = 1194
Baseline	
Age, mean ± SD, years	58.9 ± 19.4
Male sex, n (%)	618 (51.8)
Transportation to the hospital^a^, n (%)	
Critical care ambulance	376 (31.5)
Ambulance without critical care service	91 (7.6)
Family members	355 (29.7)
Self-driven	370 (31.0)
Coverage of hospitalization costs, n (%)	
Public health insurance	591 (49.5)
Complementary or private insurance	603 (50.5)
Type of ICU admission, n (%)	
Elective	317 (26.6)
Emergency	877 (73.4)
Location before transfer to the ICU, n (%)	
Emergency department	567 (47.5)
Operating room	428 (35.8)
Hospital ward	81 (6.8)
Other	118 (9.9)
Severity scores on ICU admission	
Glasgow Coma Scale, Median (IQR)	14 (9–15)
APACHE II, median (IQR)	11 (7–17)
SAPS III^b^, median (IQR)	44 (33–56)
SOFA^c^, median (IQR)	2 (1–6)
Comorbidities	
Hypertension, n (%)	603 (50.5)
Cardiopathy, n (%)	177 (14.8)
Chronic obstructive pulmonary disease, n (%)	51 (4.3)
Renal disease, n (%)	83 (7.0)
Diabetes mellitus, n (%)	218 (18.3)
Extracranial neoplasia, n (%)	89 (7.5)
Primary neurological diagnoses, n (%)	
Postoperative care of elective neurosurgery	317 (25.6)
Traumatic brain injury	218 (18,3)
Ischaemic stroke	211 (17.7)
Encephalopathy	155 (13)
Seizures	91 (7.6)
Intracerebral haemorrhage	77 (6.4)
Subarachnoid haemorrhage	70 (5.9)
Central nervous system infection	25 (2.1)
Spinal cord injury	19 (1.6)
Neuromuscular disease	11 (0.9)
Potential secondary injuries (Hs) at ICU admission	
Hypotension (MAP < 65 mmHg or SBP < 90 mmHg), n (%)	267 (22.4)
Hypoxemia (PaO_2_ < 60 mmHg or são_2_ < 90% or SpO_2_ < 90%), n (%)	72 (6.0)
Hyperthermia (body temperature > 37.5°C), n (%)	126 (10.6)
Hypercapnia (PaCO_2_ > 45 mmHg ou RR < 8 ipm), n (%)	115 (9.6)
Hypocapnia (PaCO_2_ < 35 mmHg), n (%)	213 (19.3)
Hypoglycaemia (venous or capillary glucose values < 60 mg/dL) n (%)	32 (2.7)
Hyponatremia (sodium < 135 mmHg), n (%)	200 (16.8)
Hypothermia (body temperature < 35°C), n (%)	121 (10.1)
Intracranial hypertension (intracranial pressure > 25 mmHg), n (%)	89 (7.5)
Clinical evidence of herniation, n (%)	77 (6.4)
Number of Hs, n (%)	
Zero	500 (41.9)
One	318 (26.6)
Two	203 (17.0)
Three or more	173 (14.5)
Imaging tests performed on the first day of ICU admission	
Computed tomography of the head, n (%)	931 (78)
Magnetic resonance of the head, n (%)	208 (17.4)
Cerebral arteriogram, n (%)	87 (7.3)
Procedures performed during the ICU stay	
Urgent neurosurgery, n (%)	199 (16.7)
Placement of an external ventricular drain, n (%)	84 (7.0)
Invasive mechanical ventilation, n (%)	460 (38.5)
Tracheostomy, n (%of patients placed on invasive mechanical ventilation)	146 (31.7)
Noninvasive mechanical ventilation, n (%)	77 (6.4)
Vasoactive drug—vasopressor, n (%)	358 (25.0)
Vasoactive drug—vasodilator, n (%)	82 (6.9)
Renal replacement therapy, n (%)	65 (5.4)
Intracranial pressure monitoring—intraparenchymal, n (%)	55 (4.6)
Intracranial pressure monitoring—intraventricular, n (%)	40 (3.3)
Electroencephalographic monitoring, n (%)	102 (8.5)
Intracranial Doppler monitoring, n (%)	37 (3.1)
Brain tissue oxygen pressure monitoring, n (%)	4 (0.3)
Complications	
Pneumonia, n (%)	203 (17)
Urinary tract infection, n (%)	43 (3.6)
Catheter-related infection, n (%)	35 (2.9)
Primary bloodstream infection, n (%)	27 (2.3)
Neurological infection, n (%)	23 (1.9)
Wound infection, n (%)	15 (1.2)
*Clostridium* associated diarrhoea, n (%)	2 (0.2)
Renal failure, n (%)	100 (8.4)
Acute respiratory distress syndrome, n (%)	38 (3.2)
Gastrointestinal bleeding, n (%)	15 (1.3)
Intracranial hypertension, n (%)	129 (10.8)
Outcome until the 30th study day, n (%)	
ICU discharge	832 (69.7)
Transfer to another hospital	22 (1.8)
Continued hospitalization on ICU	152 (12.7)
Mortality	188 (15.7)
Brain death^d^, n (%)	48 (25.5)
mRS on the day of the ICU outcome or until the 30th study day^e^, n (%)	
0. No symptoms at all	283 (23.7)
1. No significant disability despite symptoms; able to carry out all usual duties and activities	201 (16.9)
2. Slight disability; unable to carry out all previous activities, but able to look after own affair without assistance	121 (10.2)
3. Moderate disability; requiring some help (*e.g*., with shopping/managing affairs) but able to walk without assistance	100 (8.4)
4. Moderately severe disability; unable to walk without assistance and unable to attend to own bodily needs without assistance	154 (12.9)
5. Severe disability; bedridden, incontinent, and requiring constant nursing care and attention	140 (11.8)
6 Dead	188 (15.8)
Unfavourable outcome (mRS score 4, 5, or 6)^f^, n (%)	482 (40.6)
Length of ICU stay until the 30th study day, Median (IQR)	5 (3–16)

*APACHE II* acute physiology and chronic health evaluation, *ICU* intensive care unit, *IQR* interquartile range, *MAP* mean arterial pressure, *mRS* modified Rankin Scale, *n* absolute frequency, *PaCO*_*2*_ partial arterial carbon dioxide pressure, *PaO*_*2*_ partial arterial oxygen pressure, *RR* respiratory rate, *SaO*_*2*_ arterial oxygen saturation, *SAPS III* simplified acute physiology score III, *SBP* systolic blood pressure, *SD* standard deviation, *SOFA* sequential organ failure assessment, *SpO*_*2*_ peripheral oxygen saturation, *%* percentage within column.

^a^2 missing data.

^b^35 missing data.

^c^54 missing data.

^d^Percentage calculation considering the 188 deaths from neurological causes in the intensive care unit. In this classification, only patients who had a closed brain death protocol were considered.

^e^7 missing data.

We described these collected variables in the overall cohort and compared their rates in each of the ten diagnostic groups.

Following recommendations from observational studies in critically ill patients^9^, we determined a priori the variables to be considered prognostic factors for the two study outcomes. Thus, we evaluated the influence of age, sex, number of secondary injuries (Hs) in the first 24 h in the ICU, and GCS, APACHE II, SAPS III, and SOFA scores on these outcomes. This analysis was performed in the overall cohort and each diagnostic group.

### Analysis of disability-adjusted life-years (DALY)

We assessed the disease burden from acute neurocritical disorders using disability-adjusted life-years (DALYs) in accordance with the World Health Organization (WHO) methods and data sources for the global burden of disease estimates 2000–2015^10^ and 2000–2019^11^. One DALY represents the loss of 1 year of a "healthy" life. DALYs for a disease or health condition are calculated as the sum of the years of life lost (YLLs) due to premature mortality in the population and the years of life lost due to disability (YLDs) for individuals living with the health condition or its consequences.

The YLLs were calculated from values presented in the complete 2018 mortality table of the Brazilian Institute of Geography and Statistics (IBGE) according to sex and age as the basis for obtaining the standard life expectancy at the age at which death occurs^12^. The YLDs were estimated using disability weights available in the 2017 Global Burden of Disease (GBD) report, considering the mRS classification of patients requiring neurocritical care as parameters for selecting the disability weight^13^. The disability weight was multiplied by the prevalence of individual consequences of the disease. We estimated the population prevalence of each acute neurological disorder by sex and age range (5-year intervals) using the software DisMod-MR 2.1, which is a Bayesian meta-regression tool that was used as the main method to analyse nonfatal data in the GBD project and is recommended by WHO methodological guidelines^10,11^. For that, sample data related to prevalence rates, fatal cases, mortality, and disease remission for each acute neurocritical disorder and comorbidity relative to the total number of patients requiring neurocritical care were imputed in the software. These YLD values were also corrected for comorbidities by multiplying the weights of the sequelae of acute neurological disorders and the comorbidities presented requiring neurocritical care by each patient participating in the study.

### Statistical analysis

Categorical variables are described as absolute (n) and relative (%) frequencies, and numerical variables are described as the mean and standard deviation (SD) or median and interquartile range (IQR).

The nonparametric Mann–Whitney test was used to compare the participating ICUs in terms of the mean numbers of new patients requiring neurocritical care and ICU patients not requiring neurocritical care inpatients during the study period. Student’s t test was used to compare the mean SAPS III values and mortality rate among new patients requiring neurocritical care versus ICU patients not requiring neurocritical care an in inpatient. We compared the categorical variables between the ten diagnostic groups using the chi-square test with a subsequent two-by-two comparison with the Bonferroni correction. Given its normal distribution, age was compared between diagnostic groups using one-way analysis of variance (ANOVA) and post hoc least significant difference (LSD). Severity scores and length of ICU stay had skewed distributions and were analysed using the Kruskal–Wallis rank-sum test followed by two-by-two comparisons using Dunn’s test.

We performed univariate analysis to explore admission variables related to ICU mortality and unfavourable outcomes. The variables included in the analysis were sex, GCS, SAPS III score, SOFA score, secondary injuries (Hs) within 24 h of ICU admission, and hospitalization covered by the public health system (Brazilian Unified Health System [SUS]). Variables with statistical significance in the univariate analysis were included in the multivariate analysis. Based on results from previous studies, SAPS III was chosen over APACHE II in the multivariate models^4,14,15^. Only three multivariate models are presented, since the variables in SAPS III and SOFA overlap with those in the GCS and Hs. The results of the regression analysis were expressed as odds ratios (ORs) and 95% confidence intervals (CIs), and their statistical significance was assessed by the Wald test. The goodness of fit of the multivariate models, given by their explanatory potential, was expressed by the area under the receiver operating characteristic (ROC) curve of the model's predicted probability for the outcome. The same analysis was performed for each of the ten diagnostic subgroups.

The level of statistical significance was set at 5%. The data were analysed using the statistical software IBM SPSS, version 28.0 (SPSS Inc., Chicago, IL, USA). Missing data were not imputed.

## Results

The 36 participating sites had a median of 190 (interquartile range: 136.5 to 309.75) hospital beds and 34 (interquartile range: 20–49) ICU beds. Most participating sites were academic institutions (83.3%) located in large urban centres (63.9% in cities with > 1 million inhabitants), regardless of geographic location. The southeast region, the most populous in Brazil, had 55.5% of the participating centres, 27.8% of the centres were in the south region, 8.3% were in the northeast region and 8.3% were in the central-west region. There were no participating centres in the northern region of Brazil. Critical care physicians assisted patients in 97.2% of the 36 centres. There was a full-time neurosurgical and neurology team available in 94.4% and 83.3% of the participating hospitals, respectively. Furthermore, most centres had an active hospital protocol for treating neurological disorders. The complete profile of the participating hospitals is presented in Supplementary Table S18.

During the study period, 4245 patients were admitted to the 36 participating ICUs over 30 days (median of 98 new patients per ICU), of whom 1194 (28.1%) were patients requiring neurocritical care and 3051 (71.9%) were ICU patients not requiring neurocritical care (Supplementary Fig. S3 and Table S1).

The overall mean ICU mortality rate during the study period was 12.8% ± 8.9%. The mean mortality rate was significantly higher in patients requiring neurocritical care (17.2% ± 12.6%) than in ICU patients not requiring neurocritical care (10.1% ± 8.7%, relative risk 1.7, p = 0.038). The difference in mortality rates between neurocritical and ICU patients not requiring neurocritical care was not explained by the differences in the SAPS III results (46.9 ± 4.5 vs. 46.2 ± 12.7, respectively; p = 0.800) (Supplementary Table S1).

The study included all 1194 patients requiring neurocritical care consecutively admitted to the 36 participating ICUs over 30 days (Supplementary Fig. S3). The mean age of the patients was 58.9 ± 19.4 years and 51.8% were men. Hospitalization costs were covered by the Brazilian Unified Health System (SUS) in 49.5% (n = 591) of the cases and by complementary or private health care plans in 50.5% (n = 603) of them. The patients were divided into 10 groups according to their primary acute neurological diagnoses. The acute neurocritical condition that most often resulted in ICU admission was postoperative care of elective neurosurgery (26.5%), followed by traumatic brain injury (18.3%). The majority (58.1%) had one or more potential secondary injuries (Hs) on ICU admission. Invasive mechanical ventilation was used by 38.5% of patients, and of these, 31.7% required tracheostomy. Table 1 describes the general characteristics of the overall cohort.

The 10 diagnostic groups differed significantly in terms of patient age, sex, severity scores (GCS, APACHE II, SAPS III, and SOFA), number of potential secondary injuries (Hs) at ICU admission, percentage of patients who required invasive mechanical ventilation and tracheostomy during ICU stay, length of ICU stay, mortality, and mRS classification at the time of the outcome (30 days or day of ICU discharge) (Table 2). Supplementary Table S2 shows the comparison of other baseline characteristics of the patients, the procedures performed during their stay in the intensive care unit, complications, and the outcomes among the 10 acute neurocritical disorders, including the largest group formed of patients with postoperative care of elective neurosurgery. These variables were compared by paired diagnostic groups (pairwise comparison), as shown in Supplementary Fig. S4.

**Table 2:** Comparison of the characteristics of patients requiring neurocritical care by 10 acute neurocritical disorders.

Variables	NPO (n = 317)	TBI (n = 218)	IS (n = 211)	ENC (n = 155)	Seizures (n = 91)	ICH (n = 77)	SAH (n = 70)	SNI (n = 25)	SCI (n = 19)	NMD (n = 11)	p value
Age (years), mean ± SD	53.4 ± 14.7	54.3 ± 22.1	69.3 ± 16.9	65.7 ± 20.2	58.7 ± 22.7	62 ± 15.3	57.6 ± 14.2	55.7 ± 17.6	36.5 ± 18.5	50 ± 20.8	< 0.001^a^
Male sex, n (%)	131 (41.3)	162 (74.3)	107 (50.7)	72 (46.5)	46 (50.5)	37 (48.1)	22 (31.4)	19 (76)	14 (73.7)	8 (72.7)	< 0.001^b^
GCS, median (IQR)	15 (14–15)	9 (3–14)	14 (12–15)	13 (9–14)	14 (11–15)	10 (4–14)	10 (3–14)	10 (4–14)	15 (15—15)	15 (14—15)	< 0.001^c^
APACHE II at ICU admission, median (IQR)	7 (4–10)	14 (10–21)	11 (7–16)	15 (10–25)	10 (6–15)	15 (9–19)	14 (8–20)	20 (12–24)	7 (3–12)	7 (5–10)	< 0.001^c^
SAPS III at ICU admission^d^, median (IQR)	31 (24–39)	47 (38–57)	52 (42–58)	57 (47–68)	41 (33–54)	54.5 (44–65.5)	48 (40–60)	59 (44.5–65.5)	35 (27–43)	42 (36–49)	< 0.001^c^
SOFA at ICU admission^e^, median (IQR)	1 (0–3)	5 (2–8)	2 (0–4)	4 (2–8)	2 (0–4)	5; 4 (2–7)	4 (2–8)	6 (1.5–9)	3 (1–4)	0 (0–2)	< 0.001^c^
Number of Hs at ICU admission, n (%)											
Zero	162 (51.1)	64 (29.4)	109 (51.7)	47 (30.3)	44 (48.4)	30 (39)	24 (34.3)	6 (24)	8 (42.1)	6 (54.5)	< 0.001^b^
One	96 (30.3)	51 (23.4)	55 (26.1)	39 (25.2)	24 (26.4)	20 (26)	20 (28.6)	5 (20)	5 (26.3)	3 (27.3)	
Two	41 (12.9)	49 (22.5)	30 (14.2)	32 (20.6)	14 (15.4)	13 (16.9)	14 (20)	4 (16)	5 (26.3)	1 (9.1)	
Three or more	18 (5.7)	54 (24.8)	17 (8.1)	37 (23.9)	9 (9.9)	14 (18.2)	12 (17.1)	10 (40)	1 (5.3)	1 (9.1)	
Invasive MV, n (%)	53 (16.7)	139 (63.8)	55 (26.1)	65 (41.9)	24 (26.4)	50 (64.9)	45 (64.3)	14 (56)	12 (63.2)	3 (27.3)	< 0.001^b^
Tracheostomy, n (%)^g^	7 (13.2)	43 (30.9)	18 (32.7)	22 (33.8)	5 (20.8)	23 (46)	15 (33.3)	4 (28.6)	7 (58.3)	2 (66.7)	0.016^b^
Length of ICU stay until 30th day, median (IQR)	3 (2–5)	11 (4–29)	4 (3–11)	6 (3–17.5)	4 (2–9)	16 (6–30)	11.5 (4–22)	13 (4–26)	30 (7.5–30)	4 (3–30)	< 0.001^b^
30-day mortality, n (%)	7 (2.2)	56 (25.7)	24 (11.4)	41 (26.5)	4 (4.4)	22 (28.6)	24 (34.3)	9 (36)	1 (5.3)	0 (0)	< 0.001^b^
mRS on ICU outcome or until 30th day^f^, n (%)											
0	121 (38.3)	40 (18.3)	50 (24)	18 (11.6)	36 (40.4)	2 (2.6)	7 (10)	4 (16)	1 (5.3)	4 (36.4)	< 0.001^b^
1	75 (23.7)	30 (13.8)	37 (17.8)	16 (10.3)	16 (18)	11 (14.5)	6 (8.6)	4 (16)	4 (21.1)	2 (18.2)	
2	50 (15.8)	16 (7.3)	17 (8.2)	16 (10.3)	7 (7.9)	8 (10.5)	6 (8.6)	0 (0)	0 (0)	1 (9.1)	
3	29 (9.2)	15 (6.9)	22 (10.6)	16 (10.3)	5 (5.6)	3 (3.9)	7 (10)	1 (4)	2 (10.5)	0 (0)	
4	26 (8.2)	31 (14.2)	34 (16.3)	24 (15.5)	10 (11.2)	13 (17.1)	7 (10)	3 (12)	4 (21.1)	2 (18.2)	
5	8 (2.5)	30 (13.8)	24 (11.5)	24 (15.5)	11 (12.4)	17 (22.4)	13 (18.6)	4 (16)	7 (36.8)	2 (18.2)	
6	7 (2.2)	56 (25.7)	24 (11.5)	41 (26.5)	4 (4.5)	22 (28.9)	24 (34.3)	9 (36)	1 (5.3)	0 (0)	
Unfavourable outcome (mRS score 4, 5, or 6)^f^, n (%)	41 (13)	117 (53.7)	82 (39.4)	89 (57.4)	25 (28.1)	52 (68.4)	44 (62.9)	16 (64)	12 (63.2)	4 (36.4)	< 0.001^b^

*APACHE II* acute physiology and chronic health evaluation, *ENC* encephalopathy, *GCS* Glasgow Coma Scale, *ICH* intracerebral haemorrhage, *IQR* interquartile range, *IS* ischaemic stroke, *mRS* modified Rankin Scale, *MV* mechanical ventilation, *n* absolute frequency, *NMD* neuromuscular disease, *NPO* postoperative care of elective neurosurgery, *SAH* subarachnoid haemorrhage, *SAPS III* simplified acute physiology score III, *SCI* spinal cord injury, *SD* standard deviation, *SNI* central nervous system infection, *SOFA* sequential organ failure assessment, *TBI* traumatic brain injury, *%* percentage within column.

^**a**^Significance of the one-way analysis of variance (ANOVA).

^b^Significance of the chi-square test.

^c^Significance of the Kruskal–Wallis test.

^d^Missing data on SAPS III: 3 for NPO, 7 for TBI, 10 for STR, 1 for ENC, 1 for seizures, 5 for ICH, 5 for SAH, 1 for SNI and 2 for SCI.

^e^Missing data on SOFA: 6 for NPO, 9 for TBI, 15 for IS, 6 for seizures, 7 for ICH, 6 for SAH, 1 for SNI and 4 for SCI.

^f^Missing data on mRS and unfavourable outcome: 1 for NPO, 3 for IS, 2 for seizures, 1 for ICH.

^g^Percentage of patients placed on invasive mechanical ventilation.

The group of patients with postoperative elective neurosurgery (NPO) care accounted for 25.6% of the sample. This group, when compared to the others, had lower SAPS III (median = 31) and SOFA on admission (median = 1), in addition to a lower prevalence of potential events of secondary injuries on admission and shorter hospital stays (3 days). The comparison of this group with the others is shown in Table 2 and in the supplementary material in Fig. S4 and Table S2. The patients’ mean age was the highest and lowest in the ischaemic stroke and spinal cord injury diagnostic groups, respectively. The proportion of men was greater than 70% in the diagnostic groups of central nervous system infection, traumatic brain injury, spinal cord injury, and neuromuscular disease, and the proportion of women in the subarachnoid haemorrhage group was 68.6%. The median GCS score at ICU admission was 10 in patients with intracerebral haemorrhage, subarachnoid haemorrhage, and central nervous system infection, 9 among patients with traumatic brain injury, and ≥ 13 in patients with all other diagnoses. The APACHE II and SAPS III values were highest among patients with central nervous system infection and lowest among those admitted for postoperative care of elective neurosurgery or due to spinal cord injury. The lowest median SOFA value was observed in patients with neuromuscular disease (Table 2 and Supplementary Fig. S4).

One or more secondary injuries (Hs) at ICU admission were present in more than 50% of the patients in the following diagnostic groups: traumatic brain injury, encephalopathy, seizures, intracerebral haemorrhage, subarachnoid haemorrhage, central nervous system infection, and spinal cord injury. The group with central nervous system infection had the highest number of Hs per patient—40% of the patients with this diagnosis had three or more Hs—and the longest ICU stay (Table 2 and Supplementary Fig. S4).

More than 50% of patients with traumatic brain injury, intracerebral haemorrhage, subarachnoid haemorrhage, central nervous system infection and spinal cord injury used invasive mechanical ventilation, a proportion significantly higher than that of the postoperative care of elective neurosurgery, ischaemic stroke, and seizures groups. Among those who were intubated, the highest rate of tracheostomy occurred in the neuromuscular disease group (66.7%), followed by spinal cord injury (58.3%) and intracerebral haemorrhage (46%).

The encephalopathy group (n = 155) was composed of different aetiologies, including: 64 cases of septic encephalopathy, 26 cases of brain structural damage (hydrocephalus, tumour, etc.), 21 cases of metabolic encephalopathy, 19 cases of hypoxic-ischaemic encephalopathy, 13 cases of drug-induced encephalopathy, and 12 cases of other aetiologies. The characteristics of each of the encephalopathy aetiologies are described separately in Supplementary Table S19.

We performed univariate analysis to explore admission variables related to ICU mortality and unfavourable outcomes (mRS score 4, 5, or 6) and found that older age, lower GCS score, higher number of potential secondary injuries (Hs), admission from the emergency department, and hospitalization covered by the public health care system emerged as isolated risk factors for an unfavourable outcome (Table 3). All variables included in the univariate analysis emerged as significant and were selected for the multivariate analysis. Older age, lower GCS score, higher number of potential secondary injuries (Hs), admission from the emergency department, and hospitalization covered by the public health care system emerged as isolated risk factors for an unfavourable outcome and mortality (Table 4).

**Table 3:** Unadjusted odds ratios of prognostic factors for mortality and unfavourable outcome among patients requiring neurocritical care.

Factors (n total = 1194)	ICU discharge^a^	Death on ICU^a^	Unadjusted OR (95% CI) for ICU mortality^b^	p value^c^	Favourable outcome (mRS score 1, 2, or 3)^a^	Unfavourable outcome (mRS score 4, 5, or 6)^a^	Unadjusted OR (95% CI) for unfavourable outcome^b^	p value^c^
Age (years)	(n = 1006) 58.5 ± 19	(n = 188) 60.9 ± 21.1	1.006 (0.998–1.014)	0.122	(n = 705) 57.7 ± 18.7	(n = 482) 60.4 ± 20.2	1.007 (1.001–1.013)	0.017
Sex								
Female	519/576 (87)	75/576 (13.0)	Ref		360/572 (62,9)	212/572 (37.1)	Ref	
Male	505/618 (81.7)	113/618 (18.3)	1.494 (1.088–2.052)	0.013	345/615 (56.1)	270/615 (43.9)	1.329 (1.053–1.677)	0.017
GCS	(n = 1006) 14 (11–15)	(n = 188) 7 (3–13)	0.812 (0.784–0.840)	< 0.001	(n = 705) 15 (14–15)	(n = 482) 10 (3–14)	0.764 (0.738–0.792)	< 0.001
APACHE II	(n = 1006) 10 (6–15)	(n = 188) 21.5 (15–27.5)	1.179 (1.151–1.207)	< 0.001	(n = 705) 8 (5–12)	(n = 482)17 (12–23)	1.195 (1.168–1.222)	< 0.001
SAPS III	(n = 984) 42 (32–54)	(n = 175) 60 (47–73)	1.066 (1.054–1.078)	< 0.001	(n = 692) 39 (29–49)	(n = 460) 56 (44–67)	1.075 (1.065–1.086)	< 0.001
SOFA	(n = 968) 2 (1–4)	(n = 172) 8 (5–10)	1.366 (1.302–1.433)	< 0.001	(n = 679) 1 (0–3)	(n = 454) 6 (3–9)	1.434 (1.369–1.503)	< 0.001
Number of Hs								
Zero	478/500 (95.6)	22/500 (4.4)	Ref		380/494 (76.9)	114/494 (23.1)	Ref	
One	283/318 (89)	35/318 (11)	2.687 (1.545–4.672)	< 0.001	207/318 (65.1)	111/318 (34.9)	1.787 (1.309–2.44)	< 0.001
Two	154/203 (75.9)	49/203 (24.1)	6.91 (4.049–11.801)	< 0.001	82/202 (40.6)	120/202 (59.4)	4.878 (3.437–6.924)	< 0.001
Three or more	91/173 (52.6)	82/173 (47.4)	19.578 (11.625–32.972)	< 0.001	36/173 (20.8)	137/173 (79.2)	12.685 (8.313–19.356)	< 0.001
Coverage of hospitalization costs								
Private insurance	552/603 (91.5)	51/603 (8.5)	Ref		432/596 (72.5)	164/596 (27.5)	Ref	
Public insurance	454/591 (76.8)	137/591 (23.2)	3.266 (2.313–4.610)	< 0.001	273/591 (46.2)	318/591 (53.8)	3.068 (2.409–3.908)	< 0.001
Type of admission								
Elective	310/317 (97.8)	7/317 (2.2)	Ref		275/316 (87.0)	41/316 (13.0)	Ref	
Emergency	696/877 (77.4)	181/877 (22.6)	11.517 (5.350–24.793)	< 0.001	430/871 (49.4)	441/871 (50.6)	6.879 (4.828–9.801)	< 0.001

*APACHE II* acute physiology and chronic health evaluation II, *GCS* Glasgow Coma Scale, *Number of Hs* number of secondary injuries (resulting from the sum of the presence of hypotension, hypoxemia, hyperthermia, hypercapnia, hypocapnia, hypoglycaemia, hyponatremia, hypothermia, intracranial hypertension, and clinical evidence of herniation), *Ref.* reference category, *SAPS III* simplified acute physiology score III, *SOFA* sequential organ failure assessment.

^a^Categorical variables (sex, number of Hs, coverage of hospitalization costs, type of admission, and number of deaths) are described as absolute frequencies/total number of cases in the row (percentages), and quantitative variables are described as (number of valid cases) mean and standard deviation (age) or median and interquartile range (all other variables).

^b^Odds ratio (OR) and 95% confidence interval (95% IC) of the univariate binary logistic regression model.

^c^Wald test p value, results < 0.05 indicate statistical significance.

**Table 4:** Adjusted odds ratios of prognostic factors of mortality and unfavourable outcome for patients requiring neurocritical care.

Models	Models for ICU mortality			Models for unfavourable outcome (mRS score 4, 5, 6)		
	n	Adjusted OR (95% CI)^a^	p value^b^	n	Adjusted OR (95% CI)^a^	p value^b^
First multivariate model						
Age (years)	1194	1.022 (1.011–1.032)	0.000	1187	1.021 (1.013–1.03)	0.000
Male sex (ref: female)		1.222 (0.84–1.778)	0.295		1.03 (0.766–1.385)	0.846
GCS		0.895 (0.858–0.934)	0.000		0.835 (0.802–0.868)	0.000
Number of Hs (ref: zero Hs)						
One		1.978 (1.11–3.526)	0.021		1.259 (0.879–1.804)	0.209
Two		3.86 (2.179–6.838)	0.000		2.848 (1.889–4.294)	0.000
Three or more		8.663 (4.912–15.278)	0.000		4.761 (2.924–7.751)	0.000
Public health insurance coverage (ref: private)		2.353 (1.505–3.678)	0.000		2.784 (1.993–3.891)	0.000
Emergency admission (ref: elective)		5.77 (2.567–12.971)	0.000		4.359 (2.899–6.554)	0.000
AUC (95% CI)^c^		0.858 (0.832–0.884)	0.000		0.853 (0.832–0.875)	0.000
Second multivariate model						
Male sex (ref: female)	1194	1.184 (0.81–1.73)	0.384	1187	1.011 (0.754–1.356)	0.941
APACHE II score		1.154 (1.126–1.183)	0.000		1.161 (1.134–1.188)	0.000
Public health insurance coverage (ref: private)		2.828 (1.902–4.206)	0.000		3.383 (2.508–4.563)	0.000
Emergency admission (ref: elective)		4.726 (2.111–10.581)	0.000		4.478 (2.993–6.701)	0.000
AUC (95% CI)^c^		0.867 (0.842–0.891)	0.000		0.849 (0.827–0.871)	0.000
Third multivariate model						
Male sex (ref: female)	1159	1.431 (0.985–2.08)	0.060	1152	1.198 (0.893–1.606)	0.229
SAPS III score		1.056 (1.043–1.069)	0.000		1.07 (1.057–1.082)	0.000
Public health insurance coverage (ref: private)		3.457 (2.367–5.051)	0.000		4.851 (3.572–6.589)	0.000
Emergency admission (ref: elective)		5.455 (2.292–12.982)	0.000		3.614 (2.352–5.553)	0.000
AUC (95% CI)^c^		0.823 (0.793–0.853)	0.000		0.834 (0.81–0.857)	0.000
Fourth multivariate model						
Age (years)	1140	1.015 (1.004–1.025)	0.007	1133	1.014 (1.005–1.022)	0.001
Male sex (ref: female)		1.192 (0.811–1.752)	0.371		1.031 (0.765–1.39)	0.842
SOFA score		1.299 (1.236–1.366)	0.000		1.321 (1.258–1.386)	0.000
Public health insurance coverage (ref: private)		2.396 (1.528–3.757)	0.000		3.006 (2.144–4.214)	0.000
Emergency admission (ref: elective)		8.115 (3.277–20.098)	0.000		5.132 (3.372–7.812)	0.000
AUC (95% CI)^c^		0.855 (0.829–0.882)	0.000		0.846 (0.823–0.869)	0.000

*GCS* Glasgow Coma Scale, *ICU* intensive care unit, *mRS score* modified Rankin Scale score, *number of Hs* number of secondary injuries, *n* number of cases considered in the model, *Ref.* reference category.

^a^Odds ratio (OR) and 95% confidence interval (95% IC) of the univariate binary logistic regression model;

^b^Wald test p value, results < 0.05 indicate statistical significance.

^c^The goodness of fit of the multivariate models, given by their explanatory potential, was expressed by the area under the receiver operating characteristic curve (AUC) of the model's predicted probability for the outcome.

The same multivariate models were fitted for each diagnostic group (Supplementary Tables S3–S16) with enough cases and events to fit the models (*i.e.,* postoperative care of elective neurosurgery, traumatic brain injury, ischaemic stroke, encephalopathy, seizures, intracerebral haemorrhage, and subarachnoid haemorrhage). In subgroup analyses, older age, lower GCS, higher number of Hs, and higher APACHE II, SAPS III, and SOFA values remained consistently independent risk factors for mortality and unfavourable outcome, especially in the diagnostic groups with larger sample sizes and greater number of outcomes, where a more reliable statistical analysis was possible. Coverage of hospitalization costs by the public health care system was not a risk factor for mortality or unfavourable outcome in the diagnostic groups with traumatic brain injury, ischaemic stroke, encephalopathy, intracerebral haemorrhage, and subarachnoid haemorrhage (Supplementary Tables S6, S8, S10, S14, and S16, respectively).

**Figure 1:**
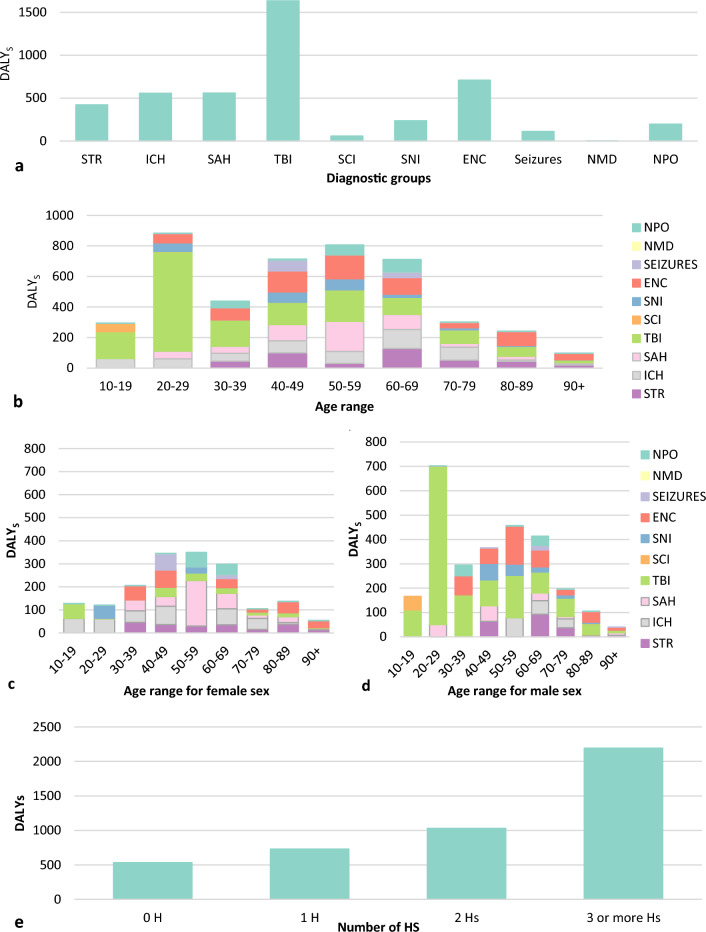
Nonstandardized DALY estimate for patients with acute neurological disorders by (**a**) primary neurological diagnoses; (**b**) age range; (**c**) age range in females; (**d**) age range in males; (**e**) number of secondary injuries (Hs). *IS* ischaemic stroke, *ICH* intracerebral haemorrhage, *SAH* subarachnoid haemorrhage, *ENC* encephalopathy, *TBI* traumatic brain injury, *SCI* spinal cord injury, *SNI* central nervous system infection, *NMD* neuromuscular disease, *NPO* postoperative care of elective neurosurgery.

Regarding disease burden, we identified a total loss of 4482.94 DALYs (4420.022 YLLs and 62.92 YLDs) in the overall cohort. The acute neurocritical disorders analysed in this study had the highest DALYs of any other condition listed for Brazil in the 2017 GBD^16^. Analysing the diagnostic groups individually, we observed that traumatic brain injury was the condition with the most years of "healthy" life lost, followed by encephalopathy and intracerebral haemorrhage (Fig. 1 and Supplementary Table S17).

The acute neurological diagnoses contributed differently to the nonstandardized DALYs in each age group. Indeed, traumatic brain injury contributed the most to the DALYs in patients between the ages of 18–39 years, while cerebrovascular diseases and encephalopathy had the greatest burden (DALYs) in patients between the ages of 40–69 years. These three diagnostic groups had a similar impact in the age groups above 70 years. When analysed by sex, the impact of traumatic brain injury and encephalopathy on DALYs was greatest in men, while that of cerebrovascular diseases (ischaemic stroke, intracerebral haemorrhage, and subarachnoid haemorrhage) was greatest in women. Furthermore, the estimated DALY was higher among patients who had a greater number of potential secondary injuries (Hs) upon admission to the ICU (Fig. 1 and Supplementary Table S17).

## Discussion

The Neurocritical Brazil Study revealed a comprehensive epidemiology of patients requiring neurocritical care and the impact of ICU admission on this patient population. These patients comprised more than one-quarter of all admissions of critically ill patients (28.1%) and had a 1.7 times higher mortality rate than ICU patients not requiring neurocritical care, a finding that was not explained by severity scores. Other studies have also shown increased mortality among patients requiring neurocritical care compared with other ICU patients^17^. The severity scores seem to discriminate to a similar degree the occurrence of severe disease in ICU patients with and without acute neurological disorders, but the reason why patients with acute neurological disorders have worse outcomes than nonneurocritical ones, despite having similar scores, is due to factors not captured in the first hours of ICU admission when the scores are measured, including longer hospital stay and a greater number of complications and nonneurological organ dysfunctions^18,19^.

The Neurocritical Brazil Study provided a better understanding of the epidemiology of patients requiring neurocritical care than we had before^20^, not only in terms of their overall and relative prevalence but also in terms of how they differ from each other in terms of demographics, clinical severity, use of ICU resources, and outcomes. Since disease burden is a measure of the prevalence and severity of a disease, it is simply not possible to estimate this measure without knowing the epidemiology of the disease^21^. Although the burden of neurological diseases has been better estimated over the past 20 years^22,23^, the burden of neurocritical diseases has remained essentially unknown until now^24^. This was the main driving force behind the design of this study, as it was critical to first know the epidemiology of patients requiring neurocritical care before estimating their burden of disease^25^.

The most common acute neurological condition for ICU admission in this study was elective neurosurgical postoperative care, which had a relatively small impact on DALYs. In contrast, patients with cerebrovascular diseases, encephalopathy, and particularly traumatic brain injury were also frequently admitted to the ICUs and imposed a substantial impact on DALYs. Traumatic brain injury is the acute critical neurological disorder with the worst impact on DALYs because it affects young patients and causes high mortality (25.7%) and a high incidence of sequelae within 30 days.. As shown in the present study, the DALYs vary significantly with the primary acute neurological diagnosis, sex, age range, and secondary injuries. We also demonstrated a clear negative impact of secondary injuries on prognosis. Of note, the increasing number of secondary injuries progressively increases the risk of a worse prognosis^26,27^. When analysed individually, each secondary neurological injury was associated with worse prognosis and increased DALYs. This indicates a clear window of opportunity to control possible secondary injuries during the first hours of the neurological injury, with an enormous beneficial impact on decreasing the burden of acute severe neurological diseases.

Acute neurocritical disorders are known to heavily burden the developing world. Despite the lack of resources for population-based health in most developing countries, there is a growing demand for resource-intensive strategies for acute neurological care^28^. The present study clearly demonstrated that acute neurocritical disorders are common in ICUs and have very high DALYs. Some strategies that could help efficiently reduce the social and economic impact of acute neurocritical disorders include increased prevention of cerebrovascular diseases, greater safety in traffic to reduce the risk of accidents, and emphasis on the prevention of potential secondary injuries to a severe primary brain injury.

Knowing how these different variables interact to exacerbate the risk of acute neurocritical disorders is essential to implement better education and improved political and social actions to minimize their negative consequences and improve results and social health. To achieve these paramount objectives, studies providing better information on the epidemiology of critically ill patients and estimating disease burden are just the beginning^29^.

All our patients were admitted to ICUs in Brazil, and different results should be expected in other countries, as demonstrated in the PRINCE Study^20,30^. In this study, it was observed that the most prevalent diagnosis on admission was subarachnoid haemorrhage, while in our study, it was postoperative elective neurosurgery followed by traumatic brain injury^20^. Associated factors, such as independent predictors of mortality, including older age, worse Glasgow Coma Scale and admission from the emergency department were also found^20^. However, compared to the epidemiological profile of the participating ICUs, we realized that we have a greater number of large urban centres when compared to other studies and, consequently, a greater number of participating academic institutions^30^. This may have influenced a different profile of neurological disorders attended.

This study has several limitations. First, the sampling of the participating centres was carried out in a nonrandom manner, and only intensive care units that were part of the network of the Brazilian Association of Intensive Care Medicine (AMIBnet) were invited to participate. Second, participation was voluntary and uncompensated, and it is possible that these factors may have affected the number of participating institutions. In addition, it is expected that among those invited, those who agreed to participate are the ICUs of hospitals that have a more developed research structure. This may have generated an overestimation of the results and may limit the generalizability of the results to all ICUs in Brazil since we cannot infer the manner in which neurocritical care is practised in other hospital settings. Third, the data collection was not monitored. We only monitored and verified incongruous data and outliers. Fourth, the sample size was determined by the number of sites and investigators who volunteered to participate and not by statistical calculations. Thus, it is possible that in the present study, the power was underestimated to detect significant differences in several of the collected variables. Fifth, the data collection took place in the mild months of August and September, and seasonal variation may have influenced the results, although these months are a transition period from winter to spring in Brazil, which must have mitigated this influence. Sixth, the follow-up of our patients was limited to 30 days and only during the ICU period; therefore, their conclusions about DALYs are limited.

Furthermore, it is known that recovery in neurocritical emergencies typically occurs over longer periods, which may overestimate YLDs based on the results of this short period among survivors. Since information about disease burden in critically ill patients is still emerging, many challenges remain to be resolved, and the understanding of these patients’ long-term outcomes is fundamental for more accurate estimates^31^. Further studies are also needed to determine which interventions and components of the ICU organization will lead to improved patient-centred outcomes^29,31^.

However, despite all these limitations, our study has several strengths. The data were collected prospectively, and the participating centres were distributed across the entire Brazilian territory, except for the North region. The study collected data from different sites across the country, which provides important insight into the global organization of neurocritical care delivery in Brazil. Additionally, most sites comprised large academic institutions located in major cities. In addition, the study provides important information about patients as well as participating centres, which enriches and strengthens the manuscript and allows the reader to assess the external validity of our study at their centre.

The collected data leave some questions to be clarified in new studies. The participation of a larger number of nonacademic institutions, including the northern region, would allow us a more complete and detailed view of the profile of the patients requiring neurocritical care in Brazil. Furthermore, understanding the activities and local practice within each participating ICU could assist in training providers to care for patients requiring neurocritical care and emergencies. All these points are of great importance, and in this study, we presented an accurate description of the epidemiology of patients requiring neurocritical care and estimated their overall and relative disease burden. These are important findings to direct policies regarding education, prevention, and treatment of severe acute neurocritical disorders.

## Conclusions

We describe a comprehensive epidemiology of patients requiring neurocritical care treated in ICUs in large urban centres in Brazil and their disease burden in the first 30 days after the acute event. Clinical, epidemiological, treatment, ICU outcomes, and DALY characteristics vary greatly with the primary acute neurological disorder. The study has great potential to guide protocols, education and health policies to minimize the adverse impact of this prevalent condition.

### Supplementary Information

Supplementary Information.

## Data Availability

The datasets generated and/or analysed during the current study are available in the Zenodo repository, https://doi.org/10.5281/zenodo.7429181.
