# Supplementary material for: Epidemiology and disease burden of patients requiring neurocritical care: a Brazilian multicentre cohort study
Source: Sci Rep. 2023 Oct 30;13:18595. doi: 10.1038/s41598-023-44261-w (PMC10616165; doi:10.1038/s41598-023-44261-w)
Supplement: Supplementary file 1 — Supplementary Information. [file 41598_2023_44261_MOESM1_ESM.pdf]

SUPPLEMENTARY MATERIAL

Epidemiology and disease burden of neurocritical disorders: a Brazilian multicentre cohort study

Álvaro Réa-Neto; Rafaella Stradiotto Bernardelli; Mirella Cristine de Oliveira; Paula Geraldes David-João; Amanda Christina Kozesinski-Nakatani; Antônio Luís Eiras Falcão; Pedro Martins Pereira Kurtz; Hélio Afonso Ghizoni Teive; Neurocritical Brazil Study group

SUPPLEMENTARY MATERIALS CONTENTS

Participating enrollment centres and investigators 5

Figure S1: Distribution of participating enrolling centres across Brazil 9

Figure S2: Percentage of contribution from each participating centre to the sample of 1194 patients 10

Figure S3: Flowchart of study enrolment. 11

Figure S4. Comparison of the characteristics of neurocritical patients admitted to the ICUs in the overall cohort and grouped by 10 neurocritical disorders. The graphs in the right column indicate the results of two-by-two (pairwise) comparisons. 12

Table S1: Overall admissions to the intensive care units and comparisons between patients with neurocritical and non-neurocritical primary diagnoses. 17

Table S2: Additional information of baseline characteristics of the patients, procedures performed during stay in the intensive care unit, complications, and outcomes in the overall cohort and in each of the 10 neurocritical diagnoses. 18

Table S3: Unadjusted odds ratios of prognostic factors for mortality and unfavourable outcome in the diagnostic group of postoperative care of elective neurosurgery in neurocritical patients admitted to intensive care units. 24

Table S4: Adjusted odds ratios of prognostic factors for mortality and Unfavourable outcome in the diagnostic group of postoperative care of elective neurosurgery in neurocritical patients admitted to intensive care units. 25

Table S5: Unadjusted odds ratios of prognostic factors for mortality and Unfavourable outcome in the diagnostic group of traumatic brain injury in neurocritical patients admitted to intensive care units. 26

Table S6: Adjusted odds ratios of prognostic factors for mortality and Unfavourable outcome in the diagnostic group of traumatic brain injury in neurocritical patients admitted to intensive care units. 27

Table S7: Unadjusted odds ratios of prognostic factors for mortality and Unfavourable outcome in the diagnostic group of ischaemic stroke in neurocritical patients admitted to intensive care units. 28

Table S8: Adjusted odds ratios of prognostic factors for mortality and Unfavourable outcome in the diagnostic group of ischaemic stroke in neurocritical patients admitted to intensive care units. 29

Table S9: Unadjusted odds ratios of prognostic factors for mortality and Unfavourable outcome in the diagnostic group of encephalopathy in neurocritical patients admitted to intensive care units. 30

Table S10: Adjusted odds ratios of prognostic factors for mortality and Unfavourable outcome in the diagnostic group of encephalopathy in neurocritical patients admitted to intensive care units. 31

Table S11: Unadjusted odds ratios of prognostic factors for mortality and Unfavourable outcome in the diagnostic group of seizures in neurocritical patients admitted to intensive care units... 32

Table S12: Adjusted odds ratios of prognostic factors for mortality and Unfavourable outcome in the diagnostic group of seizures in neurocritical patients admitted to intensive care units... 33

Table S13: Unadjusted odds ratios of prognostic factors for mortality and Unfavourable outcome in the diagnostic group of intracerebral haemorrhage in neurocritical patients admitted to intensive care units. 34

Table S14: Adjusted odds ratios of prognostic factors for mortality and Unfavourable outcome in the diagnostic group of the intracerebral haemorrhage in neurocritical patients admitted to intensive care units. 35

Table S15: Unadjusted odds ratios of prognostic factors for mortality and Unfavourable outcome in the diagnostic group of subarachnoid haemorrhage in neurocritical patients admitted to intensive care units. 36

Table S16: Adjusted odds ratios of prognostic factors for mortality and Unfavourable outcome in the diagnostic group of subarachnoid haemorrhage in neurocritical patients admitted to intensive care units. 37

Table S17: Non-standardized Disability-adjusted life-years (DALYs), years of life lost (YLLs), and years lost due to disability (YLDs) in the overall cohort of neurocritical patients and in patients grouped by main diagnosis. 38

Table S18: Characteristics of registered sites according to available resources........... 39

Table S19: Comparison of the characteristics of patients with a primary diagnosis of encephalopathy, stratified by etiologies. 41

Participating enrolment centres and investigators

Listed below are the representatives from the 36 participating intensive care units that enrolled at least one study patient. The centres are listed in order of enrollment contribution. All study sites were located in Brazil. The names of the centres are accompanied by the city and state in which they are located.

Hospital das Clínicas da Faculdade de Medicina da Universidade de São Paulo. São Paulo, São Paulo.

Fabíola Prior Caltabeloti

Hospital Beneficência Portuguesa de São Paulo. São Paulo, São Paulo.

Salomon Soriano and Viviane Cordeiro Veiga

Hospital Universitário Cajuru. Curitiba, Paraná.

Luana Alves Tannous and Juliano Gasparetto

Hospital da Bahia. Salvador, Bahia.

Fernanda Sampaio Alves

Instituto Estadual do Cérebro Paulo Niemeyer. Rio de Janeiro, Rio de Janeiro.

Pedro Martins Pereira Kurtz

Instituto de Neurologia de Curitiba. Curitiba, Paraná.

José Arthur Santos Brasil

Hospital de Base do Distrito Federal. Brasília, Distrito Federal.

Glécia Carla Rocha

Hospital Marcelino Champagnat. Curitiba, Paraná.

Jarbas Motta Junior, Bruna Martins Dzivielevski Câmara, and Livia Rodrigues Figueiredo

Hospital Copa D'Or. Rio de Janeiro, Rio de Janeiro.

Fernando Augusto Bozza, Janaína Oliveira, and William Nascimento Vianna

Hospital Municipal de Cuiabá. Cuiabá, Mato Grosso.

Diogo Roberto Lorenzo Iglesias

Hospital Vita Batel. Curitiba, Paraná.

Rafael Alexandre de Oliveira Deucher

Hospital Barra D'Or. Rio de Janeiro, Rio de Janeiro.

Fernando Augusto Bozza and Gloria Martins

Santa Casa de Misericórdia de Passos. Passos, Minas Gerais.

Marcel Resende Lopes

Fundação Hospitalar do Estado de Minas Gerais. Belo Horizonte, Minas Gerais.

Frederico Bruzzi de Carvalho

Hospital de Clínicas da Universidade Estadual de Campinas. Campinas, São Paulo.

Antônio Luis Eiras Falcão

Santa Casa São João Del Rei. São João Del Rei, Minas Gerais.

Jorge Luiz da Rocha Paranhos

Hospital de Amor. Barretos, São Paulo.

Ulysses Vasconcellos de Andrade e Silva

Hospital Caxias D'Or. Rio de Janeiro, Rio de Janeiro.

Fernando Augusto Bozza and Marco Oliveira Py

Hospital do Trabalhador. Curitiba, Paraná.

Mirella Cristine de Oliveira and Fernanda Baeumle Reese

Hospital São Lucas Copacabana. Rio de Janeiro, Rio de Janeiro.

Marcos Freitas Knibel

Hospital Estadual de Emergência e Trauma Senador Humberto Lucena. João Pessoa, Paraíba.

Gustavo Cartaxo Patriota

Hospital de Base de Rio Preto. São José do Rio Preto, São Paulo.

Suzana Margareth Ajeje Lobo and Mario Roberto Rezende Guimarães Junior

Hospital São Carlos. Fortaleza, Ceará.

Luciana de Oliveira Neves

Hospital HOME. Brasília, Distrito Federal.

Antônio Aurélio Fagundes

Hospital Israelita Albert Einstein. São Paulo, São Paulo.

Ary Serpa Neto

Hospital Novo Atibaia. Atibaia, São Paulo.

Walter Carlos Girardelli Baptista

Hospital Universitário da Universidade Estadual de Londrina. Londrina, Paraná.

Cintia Magalhães Carvalho Grion

Hospital Universitário do Oeste do Paraná. Cascavel, Paraná.

Péricles Almeida Delfino Duarte

Hospital Quinta D'Or. Rio de Janeiro, Rio de Janeiro.

Fernando Augusto Bozza and Bruno Branco

Hospital das Nações. Curitiba, Paraná.

Luísa da Silva André Salgado and Paula David João

Hospital Copa Star, Rio de Janeiro. Rio de Janeiro.

Pedro Martins Pereira Kurtz and Nívea Melo de Souza Costa

Hospital Santa Casa de Curitiba. Curitiba, Paraná.

Danilo Bastos Pompermayer and Anna Flavia Kaled

Hospital de Força Aérea do Galeão. Rio de Janeiro, Rio de Janeiro.

Rafael Brum

Hospital de Clínicas Antônio Paulino – Pronil. Nilópolis, Rio de Janeiro.

Alessandro Rocha Milan de Souza

Hospital do Câncer UOPECCAN – Filial Umuarama. Umuarama, Paraná.

Jackson Erasmo Fuck

Vitoria Apart Hospital. Serra, Espirito Santo.

Claudio Piras

Figure S1: Distribution of participating enrolling centres across Brazil

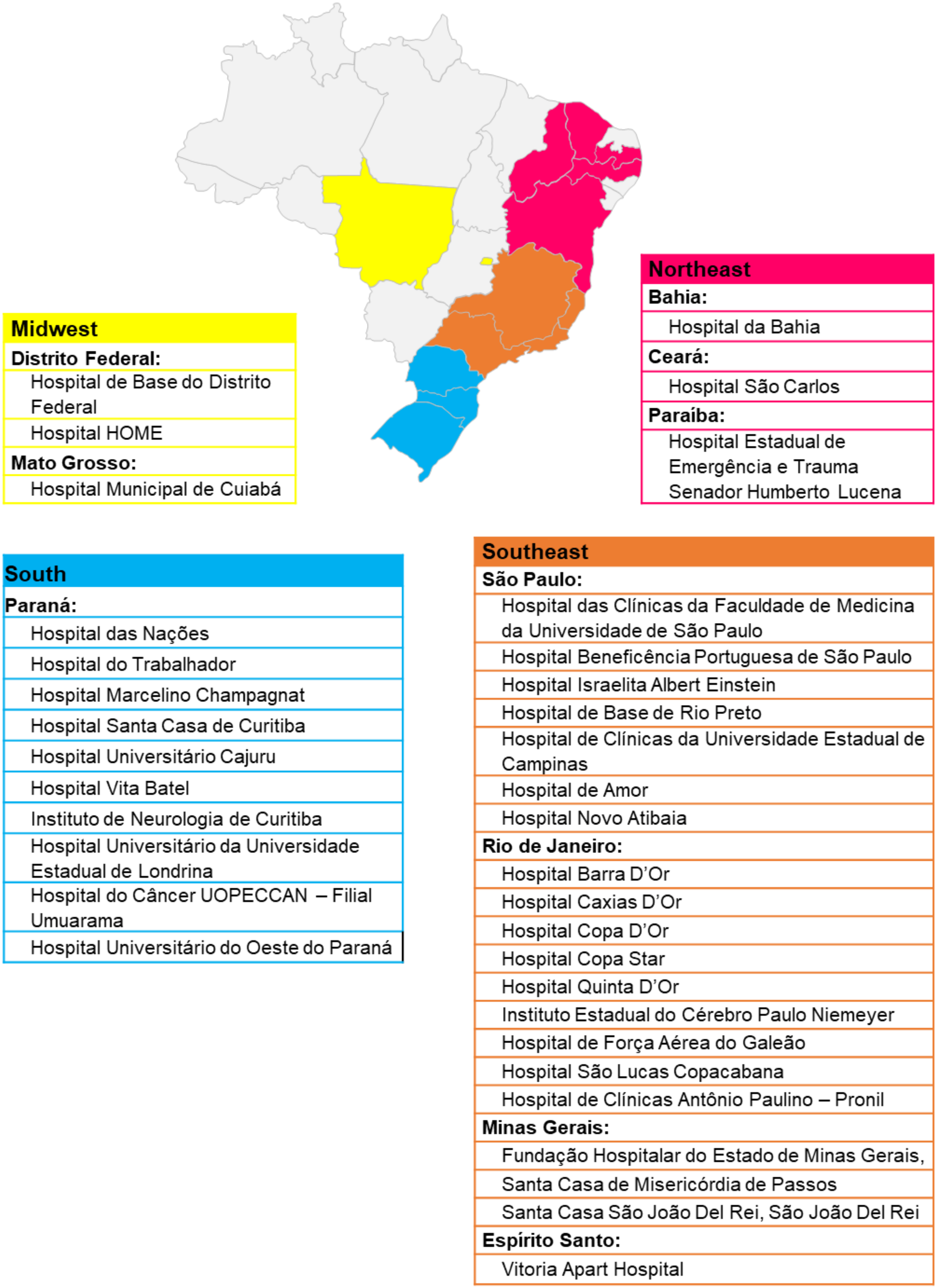

Midwest
**Distrito Federal:**
Hospital de Base do Distrito Federal
Hospital HOME
**Mato Grosso:**
Hospital Municipal de Cuiabá

Northeast
**Bahia:**
Hospital da Bahia
**Ceará:**
Hospital São Carlos
**Paraíba:**
Hospital Estadual de Emergência e Trauma Senador Humberto Lucena

South
**Paraná:**
Hospital das Nações
Hospital do Trabalhador
Hospital Marcelino Champagnat
Hospital Santa Casa de Curitiba
Hospital Universitário Cajuru
Hospital Vita Batel
Instituto de Neurologia de Curitiba
Hospital Universitário da Universidade Estadual de Londrina
Hospital do Câncer UOPECCAN – Filial Umuarama
Hospital Universitário do Oeste do Paraná

Southeast
**São Paulo:**
Hospital das Clínicas da Faculdade de Medicina da Universidade de São Paulo
Hospital Beneficência Portuguesa de São Paulo
Hospital Israelita Albert Einstein
Hospital de Base de Rio Preto
Hospital de Clínicas da Universidade Estadual de Campinas
Hospital de Amor
Hospital Novo Atibaia
**Rio de Janeiro:**
Hospital Barra D'Or
Hospital Caxias D'Or
Hospital Copa D'Or
Hospital Copa Star
Hospital Quinta D'Or
Instituto Estadual do Cérebro Paulo Niemeyer
Hospital de Força Aérea do Galeão
Hospital São Lucas Copacabana
Hospital de Clínicas Antônio Paulino – Pronil
**Minas Gerais:**
Fundação Hospitalar do Estado de Minas Gerais,
Santa Casa de Misericórdia de Passos
Santa Casa São João Del Rei, São João Del Rei
**Espírito Santo:**
Vitoria Apart Hospital

Figure S2: Percentage of contribution from each participating centre to the sample of 1194 patients

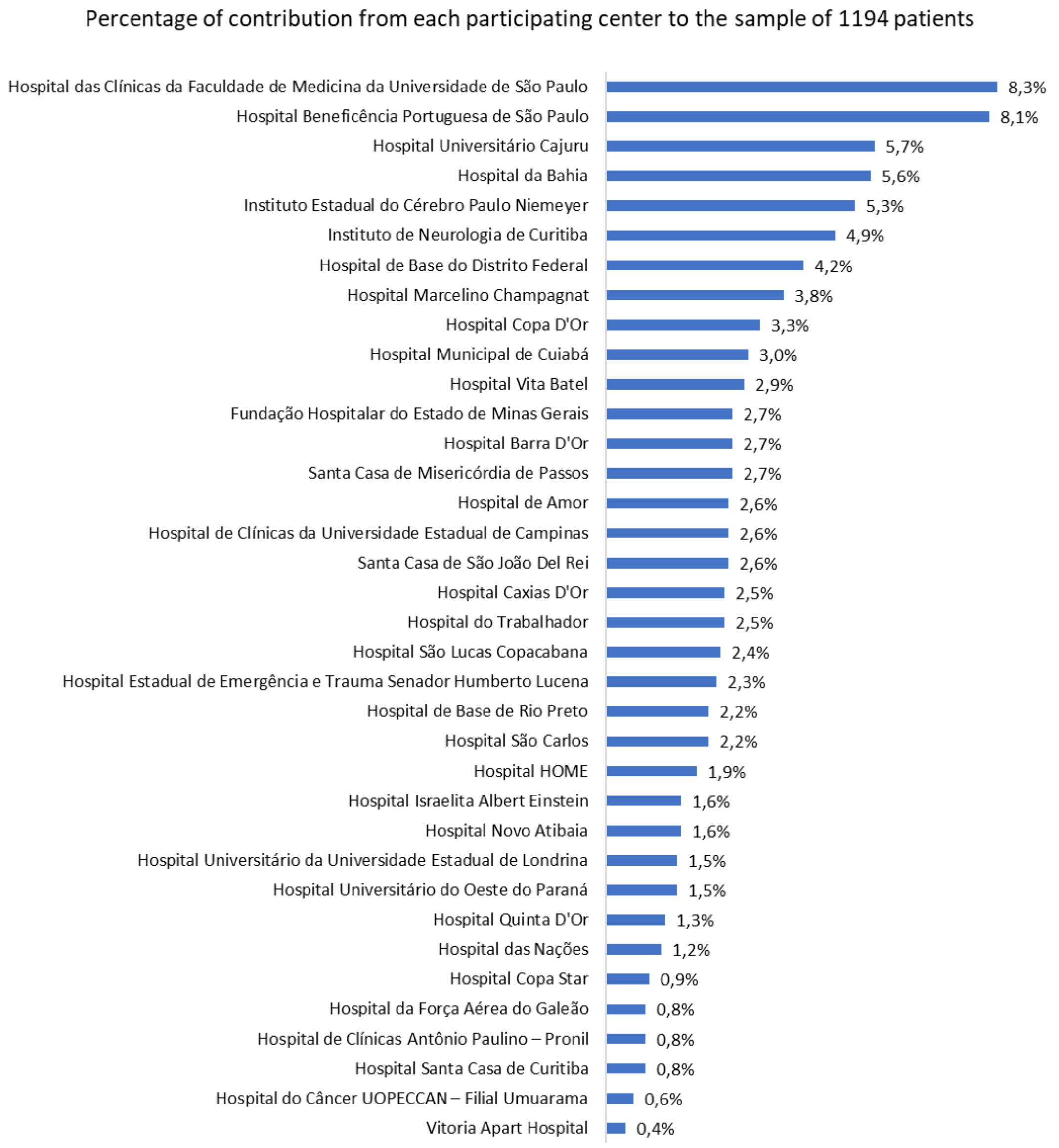

Figure S3: Flowchart of study enrolment.

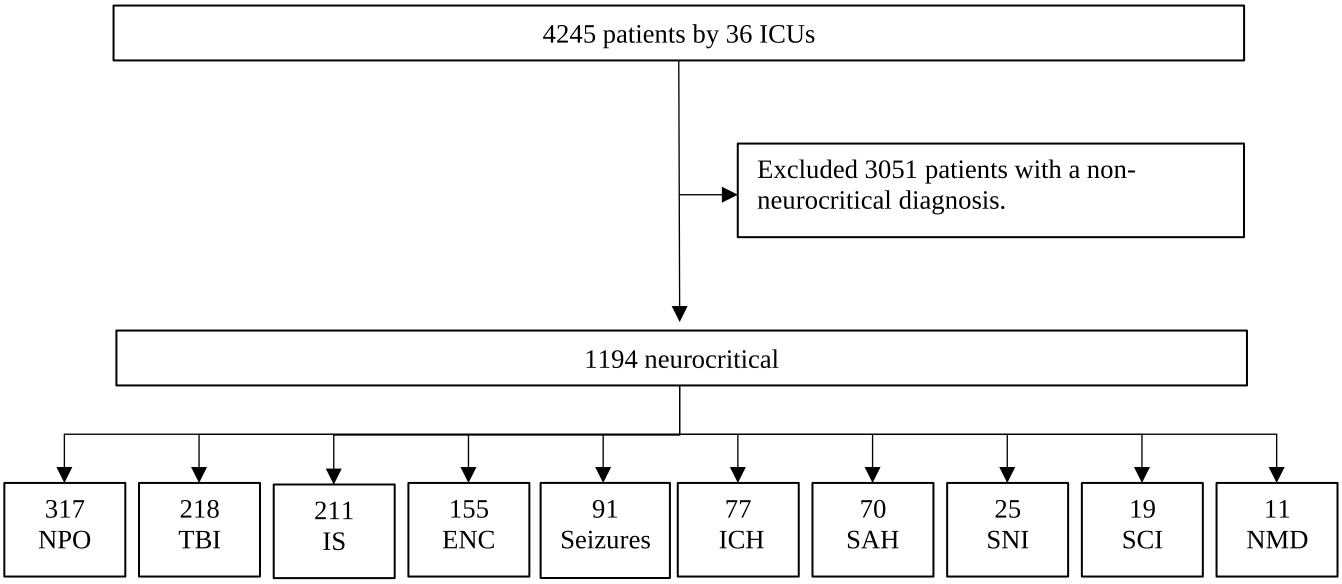

Abbreviations: ICU, intensive care unit; NPO, postoperative care of elective neurosurgery; TBI, traumatic brain injury; IS, ischaemic stroke; ENC, encephalopathy; ICH, intracerebral haemorrhage; SAH, subarachnoid haemorrhage; SNI, central nervous system infection; SCI, spinal cord injury; NMD, neuromuscular disease.

Figure S4. Comparison of the characteristics of neurocritical patients admitted to the ICUs in the overall cohort and grouped by 10 neurocritical disorders. The graphs in the right column indicate the results of two-by-two (pairwise) comparisons.

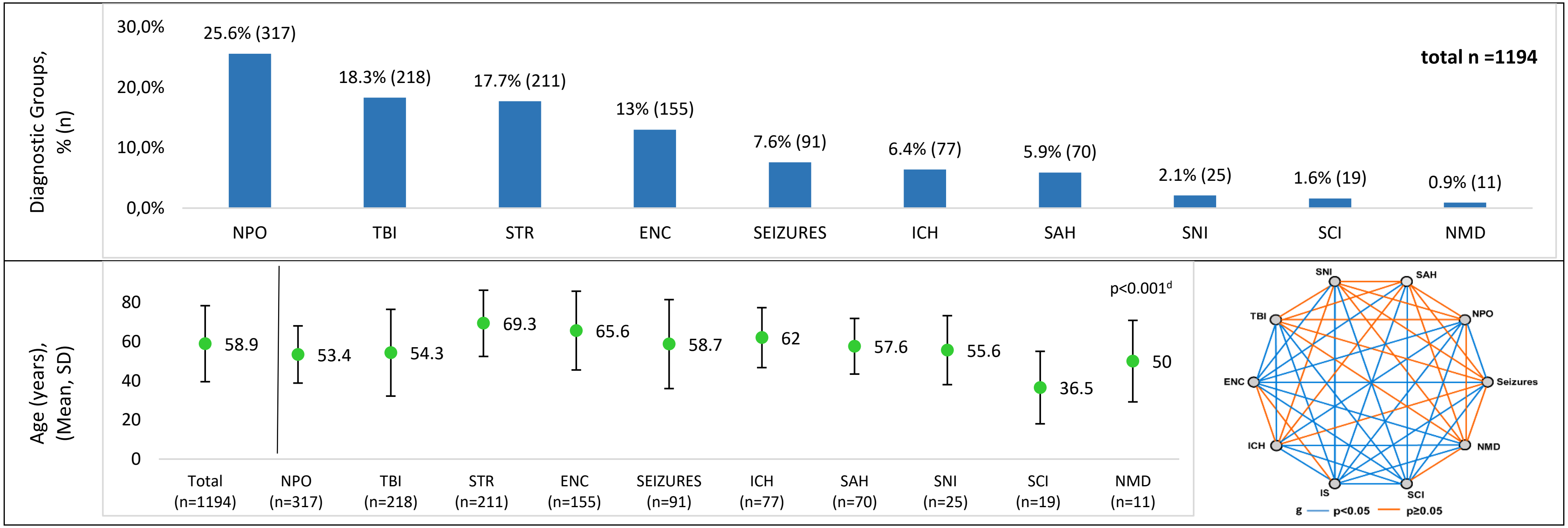

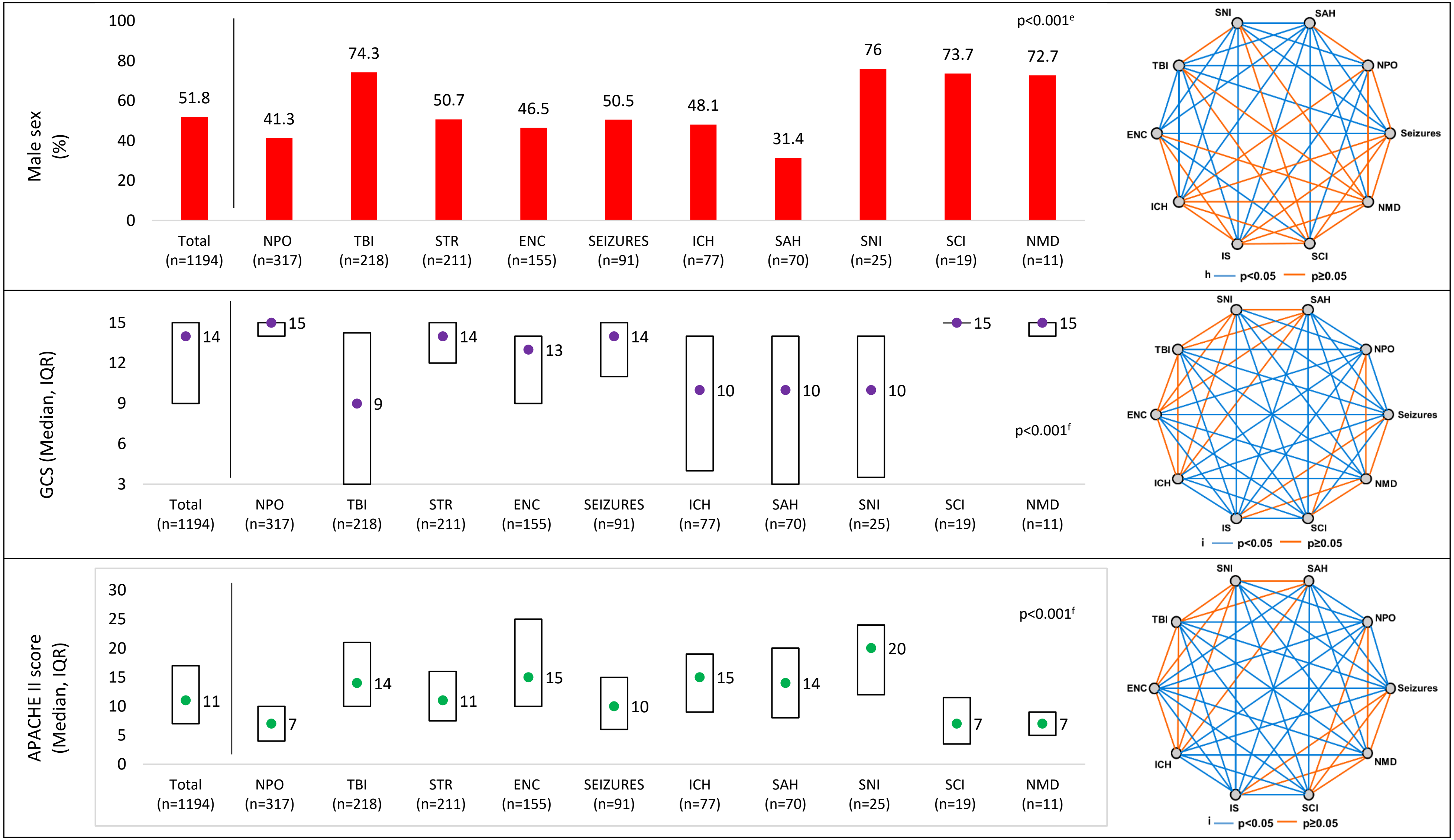
Male sex (%)
p<0.001e
Total (n=1194) 51.8
NPO (n=317) 41.3
TBI (n=218) 74.3
STR (n=211) 50.7
ENC (n=155) 46.5
SEIZURES (n=91) 50.5
ICH (n=77) 48.1
SAH (n=70) 31.4
SNI (n=25) 76
SCI (n=19) 73.7
NMD (n=11) 72.7
h p<0.05 p≥0.05
GCS (Median, IQR)
p<0.001f
Total (n=1194) 14
NPO (n=317) 15
TBI (n=218) 9
STR (n=211) 14
ENC (n=155) 13
SEIZURES (n=91) 14
ICH (n=77) 10
SAH (n=70) 10
SNI (n=25) 10
SCI (n=19) 15
NMD (n=11) 15
i p<0.05 p≥0.05
APACHE II score (Median, IQR)
p<0.001f
Total (n=1194) 11
NPO (n=317) 7
TBI (n=218) 14
STR (n=211) 11
ENC (n=155) 15
SEIZURES (n=91) 10
ICH (n=77) 15
SAH (n=70) 14
SNI (n=25) 20
SCI (n=19) 7
NMD (n=11) 7
i p<0.05 p≥0.05
SNI SAH NPO Seizures NMD SCI IS ICH ENC TBI

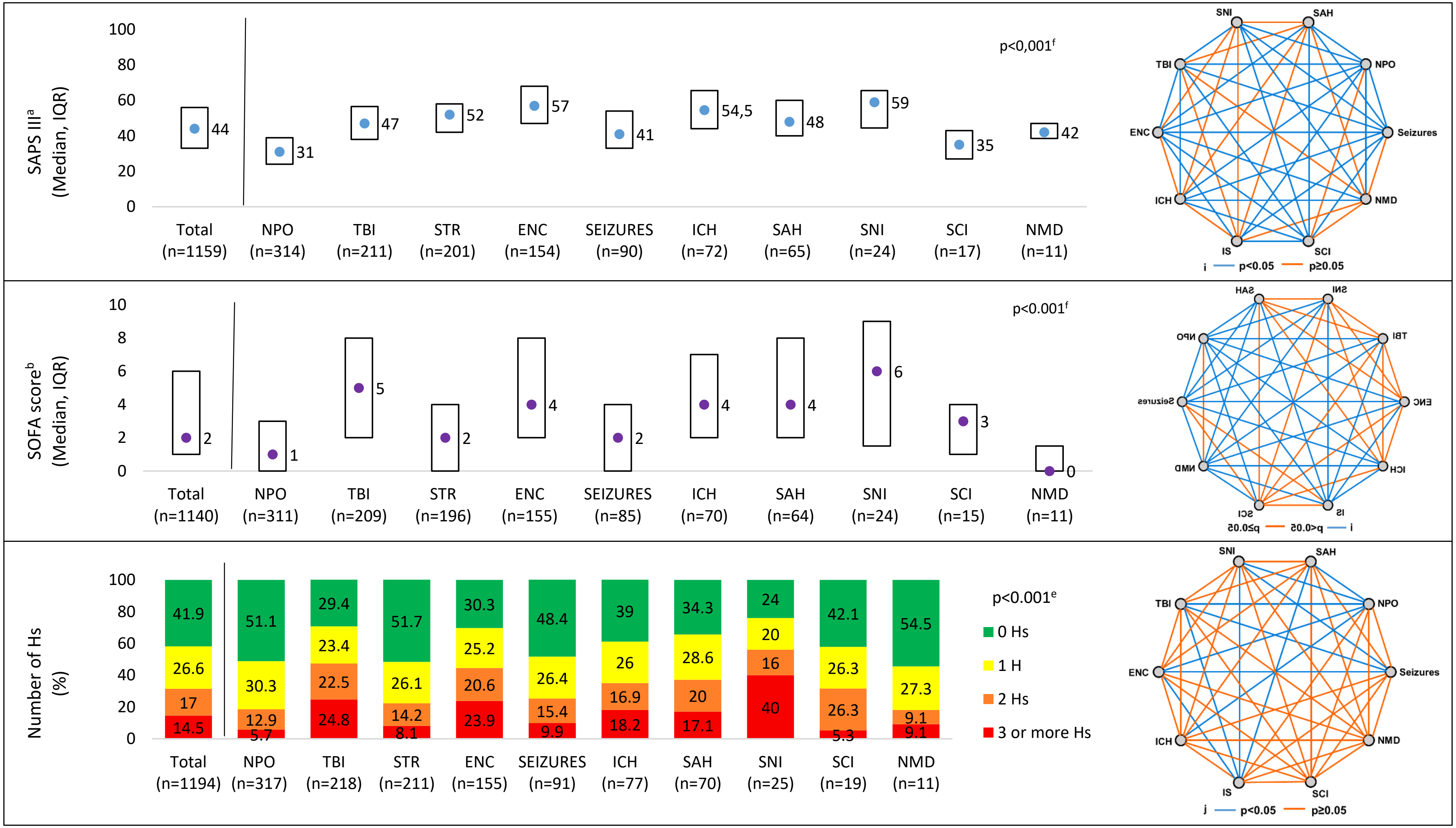
SAPS III[a] (Median, IQR)
100
80
60
40
20
0
p<0,001[f]
44
31
47
52
57
41
54,5
48
59
35
42
Total (n=1159)
NPO (n=314)
TBI (n=211)
STR (n=201)
ENC (n=154)
SEIZURES (n=90)
ICH (n=72)
SAH (n=65)
SNI (n=24)
SCI (n=17)
NMD (n=11)
SNI
SAH
TBI
NPO
ENC
Seizures
ICH
NMD
IS
SCI
i p<0.05 p≥0.05
SOFA score[b] (Median, IQR)
10
8
6
4
2
0
p<0.001[f]
2
1
5
2
4
2
4
4
6
3
0
Total (n=1140)
NPO (n=311)
TBI (n=209)
STR (n=196)
ENC (n=155)
SEIZURES (n=85)
ICH (n=70)
SAH (n=64)
SNI (n=24)
SCI (n=15)
NMD (n=11)
Number of Hs (%)
100
80
60
40
20
0
p<0.001[e]
0 Hs
1 H
2 Hs
3 or more Hs
Total (n=1194): 41.9, 26.6, 17, 14.5
NPO (n=317): 51.1, 30.3, 12.9, 5.7
TBI (n=218): 29.4, 23.4, 22.5, 24.8
STR (n=211): 51.7, 26.1, 14.2, 8.1
ENC (n=155): 30.3, 25.2, 20.6, 23.9
SEIZURES (n=91): 48.4, 26.4, 15.4, 9.9
ICH (n=77): 39, 26, 16.9, 18.2
SAH (n=70): 34.3, 28.6, 20, 17.1
SNI (n=25): 24, 20, 16, 40
SCI (n=19): 42.1, 26.3, 26.3, 5.3
NMD (n=11): 54.5, 27.3, 9.1, 9.1
SNI
SAH
TBI
NPO
ENC
Seizures
ICH
NMD
IS
SCI
j p<0.05 p≥0.05

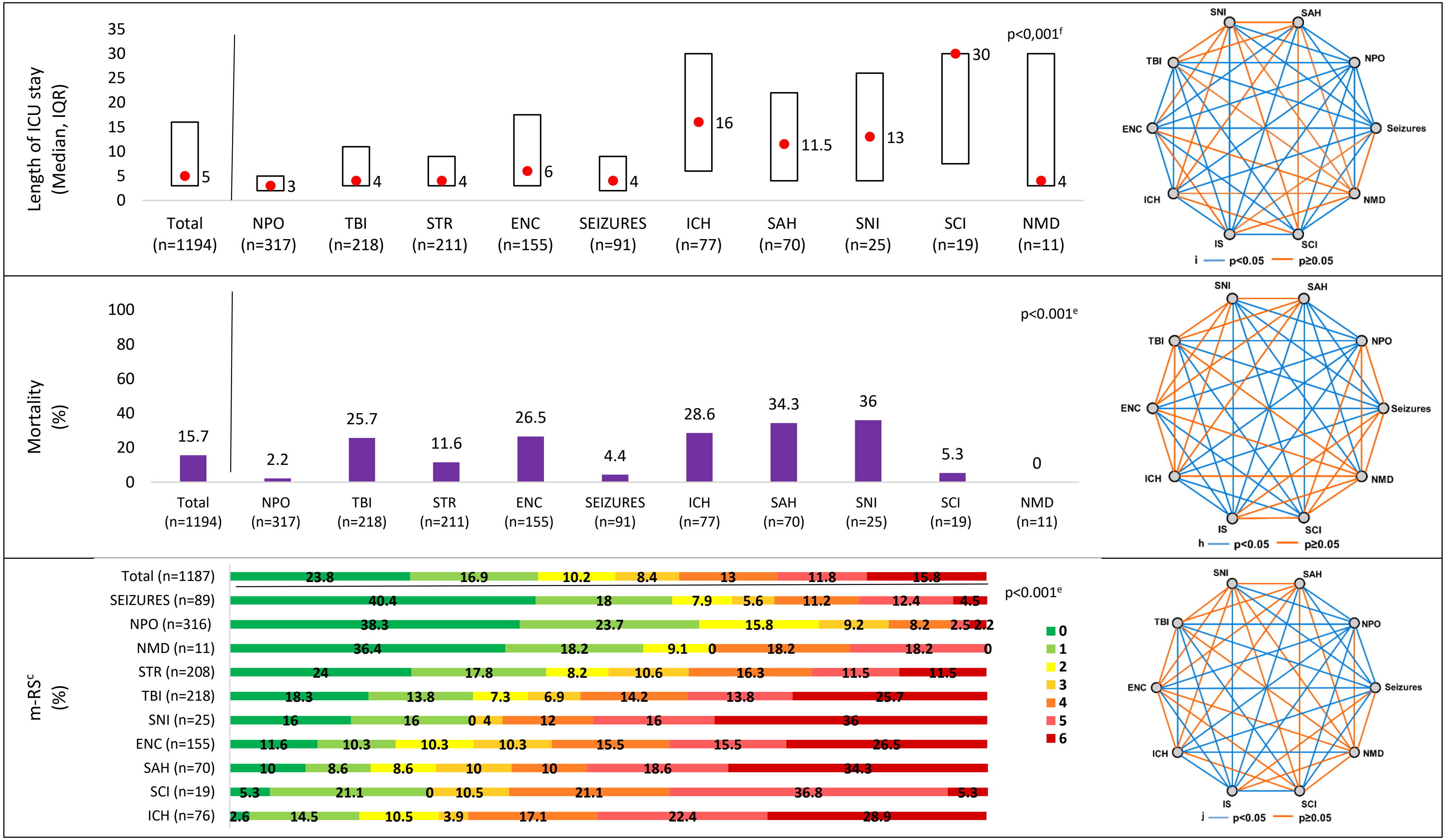
Length of ICU stay (Median, IQR)
p<0,001f
Total (n=1194) 5
NPO (n=317) 3
TBI (n=218) 4
STR (n=211) 4
ENC (n=155) 6
SEIZURES (n=91) 4
ICH (n=77) 16
SAH (n=70) 11.5
SNI (n=25) 13
SCI (n=19) 30
NMD (n=11) 4
i p<0.05 p≥0.05
Mortality (%)
p<0.001e
Total (n=1194) 15.7
NPO (n=317) 2.2
TBI (n=218) 25.7
STR (n=211) 11.6
ENC (n=155) 26.5
SEIZURES (n=91) 4.4
ICH (n=77) 28.6
SAH (n=70) 34.3
SNI (n=25) 36
SCI (n=19) 5.3
NMD (n=11) 0
h p<0.05 p≥0.05
m-RSc (%)
p<0.001e
Total (n=1187) 23.8 16.9 10.2 8.4 13 11.8 15.8
SEIZURES (n=89) 40.4 18 7.9 5.6 11.2 12.4 4.5
NPO (n=316) 38.3 23.7 15.8 9.2 8.2 2.5 2.2
NMD (n=11) 36.4 18.2 9.1 0 18.2 18.2 0
STR (n=208) 24 17.8 8.2 10.6 16.3 11.5 11.5
TBI (n=218) 18.3 13.8 7.3 6.9 14.2 13.8 25.7
SNI (n=25) 16 16 0 4 12 16 36
ENC (n=155) 11.6 10.3 10.3 10.3 15.5 15.5 26.5
SAH (n=70) 10 8.6 8.6 10 10 18.6 34.3
SCI (n=19) 5.3 21.1 0 10.5 21.1 36.8 5.3
ICH (n=76) 2.6 14.5 10.5 3.9 17.1 22.4 28.9
0 1 2 3 4 5 6
SNI SAH NPO Seizures NMD SCI IS ICH ENC TBI
j p<0.05 p≥0.05

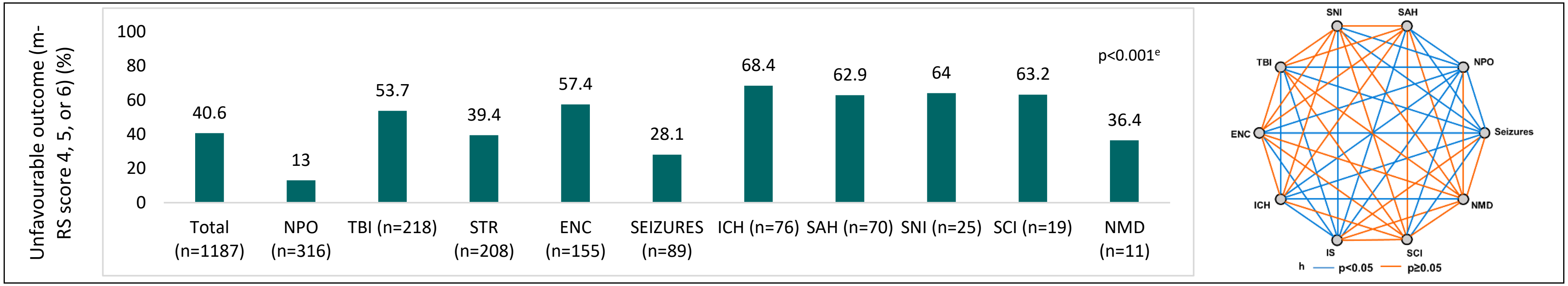

Abbreviations: NPO, postoperative care of elective neurosurgery; TBI, traumatic brain injury; IS, ischaemic stroke; ENC, encephalopathy; ICH, intracerebral haemorrhage; SAH, subarachnoid haemorrhage; SNI, central nervous system infection; SCI, spinal cord injury; NMD, neuromuscular disease; GCS, Glasgow Coma Scale; APACHE II, Acute Physiology and Chronic Health Evaluation; SAPS III, Simplified Acute Physiology Score III; SOFA, Sequential Organ Failure Assessment; m-RS, modified Rankin Scale; n, absolute frequency; %, percentage within column; IQR, interquartile range; SD, standard deviation.

[a] 35 missing data on SAPS III score; [b]54 missing data on SOFA score; [c]7 missing data on m-RS and on Unfavourable outcome.

[d] Significance level, one-way analysis of variance (ANOVA), p<0.05.

[e] Significance level, chi-square test, p<0.05.

[f] Significance level, Kruskal-Wallis test, p<0.05.

[g] Significance level, least significant difference LSD *post hoc* test, p<0.05.

[h] Significance level, Fisher's exact test, p<0.05.

[i] Significance level, Dunn's *post hoc* test, p<0.05.

[j] Significance level, chi-square's test, p<0.05.

Table S1: Overall admissions to the intensive care units and comparisons between patients with neurocritical and non-neurocritical primary diagnoses.

ICU admissions	Number of ICUs	All patients	Neurocritical patients	Non-neurocritical patients	p value
New ICU admissions in the study period, median (IQR)	30	98 (55–161)	31 (18–39)	70 (23–149)	0.013[a]
ICU mortality rate, mean ± SD	30	12.8 ± 8.9	17.2 ± 12.6	10.1 ± 8.7	0.038[b]
Mean SAPS III in the study period, mean ± SD	20	46.5 ± 7.5	46.9 ± 4.5	46.2 ± 12.7	0.800 [b]

Abbreviations: ICU, intensive care unit; IQR, interquartile range; SD, standard deviation, SAPS III, Simplified Acute Physiology Score

[a] Significance of Mann-Whitney test.

[b] Significance of independent Student *t* test.

Table S2: Additional information of baseline characteristics of the patients, procedures performed during stay in the intensive care unit, complications, and outcomes in the overall cohort and in each of the 10 neurocritical diagnoses.

Variables	Total (n=1194)	NPO (n=317)	TBI (n=218)	IS (n=211)	ENC (n=155)	Seizures (n=91)	ICH (n=77)	SAH (n=70)	SNI (n=25)	SCI (n=19)	NMD (n=11)	p value
**HOSPITALIZATION DATA**												
**Ethnicity**[c], n (%)												
White	818 (70.2)	220 (69.8)	1450 (72.5)	147 (70)	115 (74.7)	72 (79.1)	43 (55.8)	41 (60.3)	17 (68)	10 (71.4)	8 (72.7)	
Black	96 (8.2)	24 (7.6)	110 (5.5)	21 (10)	11 (7.1)	3 (3.3)	13 (16.9)	7 (10.3)	4 (16)	0 (0)	2 (18.2)	
Mulatto	26 (2.2)	5 (1.6)	70 (3.5)	5 (2.4)	1 (0.6)	1 (1.1)	3 (3.9)	3 (4.4)	1 (4)	0 (0)	0 (0)	0.051[a]
Brown	188 (16.1)	48 (15.2)	330 (16.5)	31 (14.8)	26 (16.9)	10 (11)	16 (20.8)	16 (23.5)	3 (12)	4 (28.6)	1 (9.1)	
Indigenous	2 (0.2)	0 (0)	00 (0)	1 (0.5)	0 (0)	0 (0)	1 (1.3)	0 (0)	0 (0)	0 (0)	0 (0)	
Other	35 (3)	18 (5.7)	40 (2)	5 (2.4)	1 (0.6)	5 (5.5)	1 (1.3)	1 (1.5)	0 (0)	0 (0)	0 (0)	
**Transportation to the hospital**[d], n (%)												
Critical care ambulance	376 (31.5)	7 (2.2)	134 (61.8)	47 (22.3)	46 (29.7)	20 (22)	45 (58.4)	50 (71.4)	11 (44)	15 (78.9)	1 (9.1)	
Ambulance without critical care service	91 (7.6)	15 (4.7)	20 (9.2)	15 (7.1)	14 (9)	6 (6.6)	8 (10.4)	8 (11.4)	1 (4)	1 (5.3)	3 (27.3)	<0.001[a]
Family members	355 (29.8)	100 (31.6)	37 (17.1)	89 (42.2)	56 (36.1)	37 (40.7)	17 (22.1)	6 (8.6)	7 (28)	3 (15.8)	3 (27.3)	
Self-driven	370 (31)	194 (61.4)	26 (12)	60 (28.4)	39 (25.2)	28 (30.8)	7 (9.1)	6 (8.6)	6 (24)	0 (0)	4 (36.4)	
**Source of coverage of hospital costs**, n (%)												
Complementary or private health insurance	603 (50.5)	141 (44.5)	66 (30.3)	167 (79.1)	89 (57.4)	79 (86.8)	25 (32.5)	12 (17.1)	14 (56)	2 (10.5)	8 (72.7)	<0.001[a]
Public health insurance (SUS)	591 (49.5)	176 (55.5)	152 (69.7)	44 (20.9)	66 (42.6)	12 (13.2)	52 (67.5)	58 (82.9)	11 (44)	17 (89.5)	3 (27.3)	
**Location before transfer to the ICU**, n (%)												
Emergency department	567 (47.5)	16 (5)	121 (55.5)	165 (78.2)	94 (60.6)	67 (73.6)	46 (59.7)	28 (40)	15 (60)	9 (47.4)	6 (54.5)	<0.001[a]
Operating room	428 (35.8)	281 (88.6)	73 (33.5)	13 (6.2)	14 (9)	4 (4.4)	15 (19.5)	19 (27.1)	2 (8)	7 (36.8)	0 (0)	

Hospital ward	81 (6.8)	15 (4.7)	5 (2.3)	12 (5.7)	24 (15.5)	13 (14.3)	3 (3.9)	1 (1.4)	3 (12)	1 (5.3)	4 (36.4)	
Other	118 (9.9)	5 (1.6)	19 (8.7)	21 (10)	23 (14.8)	7 (7.7)	13 (16.9)	22 (31.4)	5 (20)	2 (10.5)	1 (9.1)	
**Comorbidities**												
Hypertension, n (%)	603 (50.5)	134 (42.3)	75 (34.4)	158 (74.9)	79 (51)	37 (40.7)	56 (72.7)	46 (65.7)	10 (40)	5 (26.3)	3 (27.3)	<0.001[a]
Cardiopathy, n (%)	177 (14.8)	22 (6.9)	27 (12.4)	65 (30.8)	39 (25.2)	13 (14.3)	6 (7.8)	0 (0)	2 (8)	0 (0)	3 (27.3)	<0.001[a]
Chronic obstructive pulmonary disease, n (%)	51 (4.3)	14 (4.4)	6 (2.8)	13 (6.2)	7 (4.5)	4 (4.4)	1 (1.3)	4 (5.7)	2 (8)	0 (0)	0 (0)	0.608[a]
Renal disease, n (%)	83 (7)	15 (4.7)	11 (5)	20 (9.5)	20 (12.9)	7 (7.7)	5 (6.5)	2 (2.9)	3 (12)	0 (0)	0 (0)	0.023[a]
Diabetes, n (%)	218 (18.3)	55 (17.4)	24 (11)	74 (35.1)	27 (17.4)	14 (15.4)	13 (16.9)	6 (8.6)	1 (4)	2 (10.5)	2 (18.2)	<0.001[a]
Extracranial neoplasia, n (%)	89 (7.5)	32 (10.1)	9 (4.1)	9 (4.3)	22 (14.2)	10 (11)	3 (3.9)	0 (0)	3 (12)	0 (0)	1 (9.1)	0.001[a]
**STATUS AT ADMISSION**												
Hypotension, n (%)	267 (22.4)	57 (18)	56 (25.7)	32 (15.2)	61 (39.4)	17 (18.7)	19 (24.7)	14 (20)	7 (28)	4 (21.1)	0 (0)	<0.001[a]
Hypoxemia, n (%)	72 (6)	7 (2.2)	17 (7.8)	12 (5.7)	14 (9)	3 (3.3)	7 (9.1)	8 (11.4)	2 (8)	0 (0)	2 (18.2)	0.008[a]
Hyperthermia, n (%)	126 (10.6)	21 (6.6)	32 (14.7)	17 (8.1)	19 (12.3)	10 (11)	8 (10.4)	5 (7.1)	11 (44)	3 (15.8)	0 (0)	<0.001[a]
Hypercapnia, n (%)	115 (9.6)	14 (4.4)	46 (21.1)	8 (3.8)	17 (11)	7 (7.7)	4 (5.2)	11 (15.7)	2 (8)	4 (21.1)	2 (18.2)	<0.001[a]
Hypocapnia, n (%)	231 (19.3)	44 (13.9)	48 (22)	34 (16.1)	39 (25.2)	15 (16.5)	17 (22.1)	21 (30)	10 (40)	2 (10.5)	1 (9.1)	0.002[a]
Hypoglycaemia, n (%)	32 (2.7)	2 (0.6)	12 (5.5)	6 (2.8)	6 (3.9)	1 (1.1)	0 (0)	2 (2.9)	1 (4)	0 (0)	2 (18.2)	0.002[a]
Hyponatremia, n (%)	200 (16.8)	53 (16.7)	24 (11)	36 (17.1)	34 (21.9)	24 (26.4)	11 (14.3)	7 (10)	7 (28)	4 (21.1)	0 (0)	0.010[a]
Hypothermia, n (%)	121 (10.1)	20 (6.3)	32 (14.7)	13 (6.2)	27 (17.4)	3 (3.3)	8 (10.4)	13 (18.6)	4 (16)	1 (5.3)	0 (0)	<0.001[a]
Intracranial hypertension, n (%)	89 (7.5)	11 (3.5)	44 (20.2)	8 (3.8)	4 (2.6)	1 (1.1)	10 (13)	10 (14.3)	1 (4)	0 (0)	0 (0)	<0.001[a]
Clinical evidence of herniation, n (%)	77 (6.4)	7 (2.2)	38 (17.4)	8 (3.8)	6 (3.9)	0 (0)	11 (14.3)	5 (7.1)	1 (4)	0 (0)	1 (9.1)	<0.001[a]
**Imaging tests performed within 24h from admission**												
Computed tomography of the head , n (%)	931 (78)	195 (61.5)	194 (89)	195 (92.4)	97 (62.6)	83 (91.2)	65 (84.4)	58 (82.9)	21 (84)	19 (100)	4 (36.4)	<0.001[a]

Magnetic resonance of the head, n (%)	208 (17.4)	46 (14.5)	9 (4.1)	85 (40.3)	16 (10.3)	23 (25.3)	6 (7.8)	5 (7.1)	10 (40)	3 (15.8)	5 (45.5)	<0.001[a]
Cerebral arteriogram, n (%)	87 (7.3)	17 (5.4)	4 (1.8)	24 (11.4)	8 (5.2)	4 (4.4)	4 (5.2)	25 (35.7)	1 (4)	0 (0)	0 (0)	<0.001[a]
**PROGRESSION DATA**												
**REQUIRED PROCEDURES**												
**Emergency neurosurgery**, n (%)	199 (16.7)	18 (5.7)	81 (37.2)	14 (6.6)	7 (4.5)	4 (4.4)	34 (44.2)	30 (42.9)	4 (16)	6 (31.6)	1 (9.1)	<0.001[a]
**Placement of external ventricular drain**, n (%)	84 (7)	11 (3.5)	21 (9.6)	6 (2.8)	5 (3.2)	2 (2.2)	21 (27.3)	17 (24.3)	1 (4)	0 (0)	0 (0)	<0.001[a]
**Invasive mechanical ventilation**, n (%)	460 (38.5)	53 (16.7)	139 (63.8)	55 (26.1)	65 (41.9)	24 (26.4)	50 (64.9)	45 (64.3)	14 (56)	12 (63.2)	3 (27.3)	<0.001[a]
Tracheostomy – n (%) of patients placed on invasive mechanical ventilation	146 (31.7)	7 (13.2)	43 (30.9)	18 (32.7)	22 (33.8)	5 (20.8)	23 (46)	15 (33.3)	4 (28.6)	7 (58.3)	2 (66.7)	0.016[a]
**Noninvasive ventilation**, n (%)	77 (6.4)	10 (3.2)	16 (7.3)	13 (6.2)	17 (11)	7 (7.7)	5 (6.5)	6 (8.6)	0 (0)	1 (5.3)	2 (18.2)	0.062[a]
**VAD**, n (%)	411 (34.4)	52 (16.4)	109 (50)	55 (26.1)	61 (39.4)	13 (14.3)	49 (63.6)	49 (70)	12 (48)	10 (52.6)	1 (9.1)	<0.001[a]
Vasopressor, n (%) of all the patients who received VAD	358 (87.1)	39 (75)	106 (97.2)	42 (76.4)	60 (98.4)	12 (92.3)	35 (71.4)	41 (83.7)	12 (100)	10 (100)	1 (100)	<0.001[a]
Vasodilator, n (%) of all the patients who received VAD	82 (20)	15 (28.8)	7 (6.4)	16 (29.1)	6 (9.8)	2 (15.4)	22 (44.9)	13 (26.5)	1 (8.3)	0 (0)	0 (0)	<0.001[a]
**Dialysis**, n (%)	65 (5.4)	2 (0.6)	15 (6.9)	8 (3.8)	17 (11)	4 (4.4)	9 (11.7)	3 (4.3)	6 (24)	1 (5.3)	0 (0)	<0.001[a]
**ICP monitoring**, n (%)	96 (8)	19 (6)	39 (17.9)	5 (2.4)	2 (1.3)	1 (1.1)	15 (19.5)	14 (20)	1 (4)	0 (0)	0 (0)	<0.001[a]
Intraparenchymal, n (%) of all the patients who underwent ICP monitoring	55 (57.3)	14 (73.7)	30 (76.9)	3 (60)	0 (0)	0 (0)	5 (33.3)	2 (14.3)	1 (100)	0 (0)	0 (0)	<0.001[a]
Intraventricular, n (%) of all the patients who underwent ICP monitoring	40 (41.7)	5 (26.3)	8 (20.5)	2 (40)	1 (50)	1 (100)	10 (66.7)	12 (85.7)	1 (100)	0 (0)	0 (0)	<0.001[a]
**Other monitoring measures**, n (%)	121 (10.1)	7 (2.2)	24 (11)	28 (13.3)	7 (4.5)	32 (35.2)	9 (11.7)	12 (17.1)	2 (8)	0 (0)	0 (0)	<0.001[a]

EEG, n (%) of all the patients who received other monitoring measures	102 (84.3)	6 (85.7)	16 (66.7)	25 (89.3)	7 (100)	32 (100)	9 (100)	5 (41.7)	2 (100)	0 (0)	0 (0)	<0.001[a]
Intracranial Doppler, n (%) of all the patients who received other monitoring measures	4 (3.3)	2 (28.6)	1 (4.2)	0 (0)	0 (0)	0 (0)	1 (11.1)	0 (0)	0 (0)	0 (0)	0 (0)	<0.001[a]
$PtiO_2$, n (%) of all the patients who received other monitoring measures	37 (30.6)	3 (42.9)	12 (50)	6 (21.4)	0 (0)	3 (9.4)	2 (22.2)	11 (91.7)	0 (0)	0 (0)	0 (0)	0.010[a]
**Developed infection**, n (%)	292 (24.5)	19 (6)	83 (38.1)	52 (24.6)	41 (26.5)	15 (16.5)	34 (44.2)	24 (34.3)	12 (48)	11 (57.9)	1 (9.1)	<0.001[a]
Pneumonia, n (%) of all the patients who developed infection	203 (69.3)	11 (57.9)	62 (74.7)	36 (67.9)	29 (70.7)	8 (53.3)	25 (73.5)	20 (83.3)	6 (50)	6 (54.5)	0 (0)	0.204[a]
Urinary infection, n (%) of all the patients who developed infection	43 (14.7)	4 (21.1)	8 (9.6)	13 (24.5)	7 (17.1)	4 (26.7)	3 (8.8)	1 (4.2)	2 (16.7)	1 (9.1)	0 (0)	0.222[a]
Catheter-related infection, n (%) of all the patients who developed infection	35 (11.9)	2 (10.5)	12 (14.5)	2 (3.8)	2 (4.9)	2 (13.3)	8 (23.5)	1 (4.2)	4 (33.3)	2 (18.2)	0 (0)	0.043[a]
Primary bacterial infection, n (%) of all the patients who developed infection	27 (9.2)	0 (0)	8 (9.6)	5 (9.4)	4 (9.8)	4 (26.7)	3 (8.8)	1 (4.2)	1 (8.3)	1 (9.1)	0 (0)	0.507[a]
Neurological infection, n (%) of all the patients who developed infection	23 (7.8)	5 (26.3)	4 (4.8)	1 (1.9)	1 (2.4)	1 (6.7)	1 (2.9)	2 (8.3)	7 (58.3)	1 (9.1)	0 (0)	<0.001[a]
Surgical wound infection, n (%) of all the patients who developed infection	15 (5.1)	1 (5.3)	2 (2.4)	5 (9.4)	2 (4.9)	0 (0)	1 (2.9)	2 (8.3)	0 (0)	1 (9.1)	1 (100)	0.004[a]
Clostridium associated diarrhea, n (%) of all the patients who developed infection	2 (0.7)	0 (0)	0 (0)	0 (0)	0 (0)	2 (13.3)	0 (0)	0 (0)	0 (0)	0 (0)	0 (0)	<0.001[a]
**Renal failure**, n (%)	100 (8.4)	3 (0.9)	22 (10.1)	16 (7.6)	26 (16.8)	4 (4.4)	16 (20.8)	6 (8.6)	5 (20)	2 (10.5)	0 (0)	<0.001[a]

**Acute respiratory distress syndrome**, n (%)	38 (3.2)	4 (1.3)	14 (6.4)	3 (1.4)	3 (1.9)	2 (2.2)	3 (3.9)	3 (4.3)	2 (8)	4 (21.1)	0 (0)	<0.001[a]
**Gastrointestinal bleeding,** n (%)	15 (1.3)	1 (0.3)	3 (1.4)	2 (0.9)	3 (1.9)	2 (2.2)	1 (1.3)	1 (1.4)	1 (4)	0 (0)	1 (9.1)	0.283[a]
**IH**, n (%)	129 (10.8)	14 (4.4)	56 (25.7)	8 (3.8)	6 (3.9)	2 (2.2)	21 (27.3)	19 (27.1)	3 (12)	0 (0)	0 (0)	<0.001[a]
Intraparenchymal monitoring in IH, n (%) of all patients who underwent IH	30 (23.3)	8 (57.1)	14 (25)	1 (12.5)	1 (16.7)	0 (0)	4 (19)	2 (10.5)	0 (0)	0 (0)	0 (0)	0.067[a]
IH treatment with mannitol, n (%) of all patients who underwent IH	82 (63.6)	12 (85.7)	32 (57.1)	4 (50)	3 (50)	1 (50)	14 (66.7)	15 (78.9)	1 (33.3)	0 (0)	0 (0)	0.295[a]
IH treatment with decompressive craniectomy, n (%) of all patients who underwent IH	41 (31.8)	5 (35.7)	19 (33.9)	5 (62.5)	0 (0)	0 (0)	7 (33.3)	4 (21.1)	1 (33.3)	0 (0)	0 (0)	0.294[a]
IH treatment with external ventricular drainage, n (%) of all patients who underwent IH	46 (35.7)	5 (35.7)	13 (23.2)	1 (12.5)	2 (33.3)	1 (50)	13 (61.9)	10 (52.6)	1 (33.3)	0 (0)	0 (0)	0.042[a]
IH treatment with surgical drainage, n (%) of all patients who underwent IH	71 (55)	6 (42.9)	39 (69.6)	0 (0)	2 (33.3)	0 (0)	15 (71.4)	7 (36.8)	2 (66.7)	0 (0)	0 (0)	0.001[a]
IH treatment with barbiturate coma therapy, n (%) of all patients who underwent IH	10 (7.8)	1 (7.1)	5 (8.9)	1 (12.5)	0 (0)	1 (50)	1 (4.8)	1 (5.3)	0 (0)	0 (0)	0 (0)	0.478[a]
**OUTCOME**												
**Length of ICU stay until 30th day among survivors**[e], median (IQR)	4 (2 - 9)	3 (1 - 5)	8 (3 - 18)	4 (2 - 8)	5 (3 - 12)	3.5 (2 - 6)	8.5 (5 - 19)	9 (4 - 14)	4 (3 - 14)	7 (5 - 10)	4 (2 - 4)	<0.001[b]
**Length of ICU stay until 30th day among the deceased**[f], median (IQR)	8.5 (4 - 15)	6 (5 - 18)	8 (3 - 13.5)	9 (4.5 - 13.5)	5 (2 - 15)	11 (9.5 - 12)	9.5 (4 - 18)	8.5 (4 - 14)	13 (11 - 23)	No deaths until 30th day	No deaths until 30th day	0.250[b]
**30-day outcome**, n (%)												<0.001[a]
Discharge	832 (69.7)	300 (94.6)	113 (51.8)	166 (78.7)	92 (59.4)	75 (82.4)	30 (39)	30 (42.9)	10 (40)	9 (47.4)	7 (63.6)	

Transference	22 (1.8)	0 (0)	5 (2.3)	3 (1.4)	4 (2.6)	3 (3.3)	2 (2.6)	4 (5.7)	1 (4)	0 (0)	0 (0)	
Death without limitation of life support	117 (9.8)	6 (1.9)	32 (14.7)	15 (7.1)	20 (12.9)	3 (3.3)	15 (19.5)	18 (25.7)	7 (28)	1 (5.3)	0 (0)	
Death with limitation of life support	71 (5.9)	1 (0.3)	24 (11)	9 (4.3)	21 (13.5)	1 (1.1)	7 (9.1)	6 (8.6)	2 (8)	0 (0)	0 (0)	
Continued hospitalization (patient room)	75 (6.3)	5 (1.6)	23 (10.6)	10 (4.7)	8 (5.2)	5 (5.5)	11 (14.3)	5 (7.1)	3 (12)	2 (10.5)	3 (27.3)	
Continued ICU hospitalization	77 (6.4)	5 (1.6)	21 (9.6)	8 (3.8)	10 (6.5)	4 (4.4)	12 (15.6)	7 (10)	2 (8)	7 (36.8)	1 (9.1)	
**Cause of death**												
Death due to neurological cause[f], n (%) of the total deaths	100 (53.2)	6 (85.7)	38 (67.9)	10 (41.7)	7 (17.1)	2 (50)	14 (63.6)	20 (83.3)	3 (33.3)	0 (0)	0 (0)	<0.001[a]
Brain death protocol[h], n (%) of the total deaths due to neurological cause	48 (48)	3 (50)	27 (71.1)	2 (20)	2 (28.6)	0 (0)	4 (28.6)	9 (45)	1 (33.3)	0 (0)	0 (0)	0.020[a]

Abbreviations: NPO, postoperative care of elective neurosurgery; TBI, traumatic brain injury; IS, ischaemic stroke; ENC, encephalopathy; ICH, intracerebral haemorrhage; SAH, subarachnoid haemorrhage; SNI, central nervous system infection; SCI, spinal cord injury; NMD, neuromuscular disease; ICU, intensive care unit; SUS, Brazilian Unified Health System; VAD, vasoactive drug, ICP, intracranial pressure; EEG, electroencephalogram; $PtiO_2$, brain tissue oxygen pressure; IH, intracranial hypertension; m-RS, modified Rankin scale score; n, absolute frequency; %, percentage within column; IQR, interquartile range; SD, standard deviation.

[a] Significance of the chi-square test.

[b] Significance of the Kruskal-Wallis test.

[c] Missing data on Ethnicity: 29 on Total; 2 on NPO, 18 on TBI, 1 on IS, 1 on ENC, 2 on SAH and 5 on SCI.

[d] Missing data on transportation to the hospital: 2 on Total; 1 on NPO, 1 on TBI.

[e] Considering only those discharged from the ICU, which are: 832 on Total, 300 on NPO, 113 on TBI, 166 on IS, 92 on ENC, 75 on Seizures, 30 on ICH, 30 on SAH, 10 on SNI, 9 on SCI, and 7 on NMD.

[f] Considering only those who died in the ICU until 30th day, which are: 187 on Total, 7 on NPO, 56 on TBI, 24 on IS, 41 on ENC, 4 on Seizures, 22 on ICH, 24 on SAH, 9 on SNI, 0 on SCI, and 0 on NMD.

[g] Considering only deaths due to neurological cause until 30th day, which are: 100 on Total, 6 on NPO, 38 on TBI, 10 on IS, 7 on ENC, 2 on Seizures, 14 on ICH, 20 on SAH, 3 on SNI, 0 on SCI, and 0 on NMD.

Table S3: Unadjusted odds ratios of prognostic factors for mortality and unfavourable outcome in the diagnostic group of **postoperative care of elective neurosurgery** in neurocritical patients admitted to intensive care units.

Factors	ICU discharge[a]	Death on ICU[a]	Unadjusted OR (95% CI) for ICU Mortality[b]	p value[c]	Favourable outcome (m-RS score 1, 2, or 3)[a]	Unfavourable outcome (m-RS score 4, 5, or 6)[a]	Unadjusted OR (95% CI) for Unfavourable outcome[b]	p value[c]
Age (years)	(n=310) 53.4 ± 14.7	(n=7) 56.6 ± 11.4	1.015 (0.964–1.069)	0.566	(n=275) 52.7 ± 14.5	(n=41) 57.8 ± 15	1.024 (1.001–1.048)	0.040
Sex								
Female	182/186 (97.8)	4/186 (2.2)	Ref.		134/141 (100)	7/141 (0)	Ref.	
Male	128/131 (97.7)	3/131 (2.3)	1.066 (0.235–4.846)	0.934	169/176 (93)	7/176 (7)	1.599 (0.828–3.089)	0.162
Glasgow Coma Scale	(n=310) 15 (14–15)	(n=7) 14 (7–15)	0.826 (0.702–0.971)	0.021	(n=275) 15 (14–15)	(n=41) 15 (12–15)	0.889 (0.805–0.982)	0.020
APACHE II	(n=310) 7 (4–10)	(n=7) 13 (9–17)	1.196 (1.062–1.347)	0.003	(n=275) 7 (4–10)	(n=41) 10 (6–14)	1.106 (1.041–1.176)	0.001
SAPS III	(n=308) 31.0 (24–39)	(n=6) 40.5 (32–68)	1.087 (1.03–1.148)	0.002	(n=274) 30 (24–37)	(n=39) 34 (31–46)	1.054 (1.025–1.083)	0.000
SOFA	(n=305) 1 (0–2)	(n=6) 5 (1–8)	1.193 (1.024–1.39)	0.024	(n=271) 1 (0–2)	(n=39) 3 (0–5)	1.163 (1.052–1.284)	0.003
Number of Hs								
Zero	160/162 (98.8)	2/162 (1.2)	Ref.		134/141 (100)	7/141 (0)	Ref.	
One	95/96 (99)	1/96 (1)	0.842 (0.075–9.412)	0.889	169/176 (93)	7/176 (7)	1.186 (0.487–2.888)	0.708
Two	40/41 (97.6)	1/41 (2.4)	2 (0.177–22.612)	0.575	134/141 (100)	7/141 (0)	3.821 (1.533–9.519)	0.004
Three or more	15/18 (83.3)	3/18 (16.7)	16 (2.476–103.381)	0.004	169/176 (93)	7/176 (7)	11.462 (3.877–33.884)	0.000
Coverage of hospitalization costs								
Private insurance	134/141 (100)	7/141 (0)			133/140 (95)	7/140 (5)	Ref.	
Public insurance	169/176 (93)	7/176 (7)	N/A	N/A	142/176 (80.7)	34/176 (19.3)	4.549 (1.95–10.614)	0.000

[a] Categorical variables are described as the absolute number of deaths (percentage of deaths by the total number of cases in the line); age is described as mean ± standard deviation, and the other quantitative variables are described as median (first quartile – third quartile).
[b] Odds ratios and 95% confidence intervals of the univariate binary logistic regression model.
[c] Wald test p value, $p < 0.05$ indicate statistical significance.
Abbreviations: ICU, intensive care unit; m-RS, modified Ranking scale score; OR, odds ratio; ref., reference; APACHE II, Acute Physiology and Chronic Health Evaluation II; SAPS III, Simplified Acute Physiology Score III; SOFA, Sequential Organ Failure Assessment; Hs, number of secondary injuries, resulting from the sum of the presence of hypotension, hypoxemia, hyperthermia, hypercapnia, hypocapnia, hypoglycaemia, hyponatremia, hypothermia, intracranial hypertension, and clinical evidence of herniation; N/A, not analysed.

Table S4: Adjusted odds ratios of prognostic factors for mortality and Unfavourable outcome in the diagnostic group of **postoperative care of elective neurosurgery** in neurocritical patients admitted to intensive care units.

**Factors**	**Models for ICU mortality**		**Models for Unfavourable outcome (m-RS 4, 5, or 6)**	
**First multivariate model**	**Adjusted OR (95% CI)[b]**	**p value [c]**	**Adjusted OR (95% CI)[b]**	**p value [c]**
Age (years)	1.021 (0.96–1.087)	0.510	1.033 (1.006–1.06)	0.014
Male sex (ref. female)	1.262 (0.245–6.505)	0.781	2.067 (0.985–4.339)	0.055
Glasgow Coma Scale	0.864 (0.708–1.054)	0.150	0.919 (0.816–1.034)	0.161
Number of Hs (ref. zero Hs)				
One	0.638 (0.055–7.425)	0.719	0.963 (0.382–2.429)	0.936
Two	1.549 (0.132–18.15)	0.727	3.5 (1.343–9.122)	0.010
Three or more	6.649 (0.852–51.859)	0.071	7.229 (2.202–23.734)	0.001
Public health insurance coverage (ref. private)			5.087 (2.026–12.775)	0.001
N included in the model[a]	317		316	
AUC (95% CI) [d]	0.924 (0.861–0.987)	0.000	0.800 (0.731–0.87)	0.000
**Second multivariate model**	**Adjusted OR (95% CI)[b]**	**p value [c]**	**Adjusted OR (95% CI)[b]**	**p value [c]**
Male sex (ref. female)	1.046 (0.212–5.149)	0.956	1.737 (0.872–3.46)	0.116
APACHE II score	1.191 (1.047–1.355)	0.008	1.098 (1.029–1.171)	0.004
Public health insurance coverage (ref. private)			4.331 (1.832–10.239)	0.001
N included in the model[†]	317		316	
AUC (95% CI) [d]	0.881 (0.812–0.95)	0.001	0.737 (0.67–0.804)	0.000
**Third multivariate model**	**Adjusted OR (95% CI)[b]**	**p value [c]**	**Adjusted OR (95% CI)[b]**	**p value [c]**
Male sex (ref. female)	0.942 (0.155–5.73)	0.948	1.886 (0.922–3.859)	0.082
SAPS III score	1.083 (1.025–1.145)	0.005	1.057 (1.026–1.088)	0.000
Public health insurance coverage (ref. private)			4.53 (1.89–10.856)	0.001
N included in the model[†]	314		313	
AUC (95% CI) [d]	0.85 (0.726–0.974)	0.003	0.759 (0.687–0.832)	0.000
**Fourth multivariate model**	**Adjusted OR (95% CI)[b]**	**p value [c]**	**Adjusted OR (95% CI)[b]**	**p value [c]**
Age (years)	1.013 (0.951–1.08)	0.687	1.028 (1.002–1.055)	0.033
Male sex (ref. female)	0.754 (0.13–4.392)	0.754	1.877 (0.923–3.817)	0.082
SOFA score	1.147 (0.978–1.346)	0.092	1.119 (1.008–1.242)	0.035
Public health insurance coverage (ref. private)			4.264 (1.753–10.372)	0.001
N included in the model[†]	311		310	
AUC (95% CI) [d]	0.825 (0.699–0.952)	0.006	0.756 (0.684–0.827)	0.000

[a] Number of cases (n) included in the multivariate model. [b]Odds ratios and 95% confidence intervals of the multivariate binary logistic regression model. [c] Wald test p value, results < 0.05 indicate statistical significance. [d]Area under the receiver operating characteristic curve and 95% confidence interval. Abbreviations: ICU, intensive care unit; m-RS, modified Rankin scale score; 95% CI, 95% confidence interval; ref., reference; APACHE II, Acute Physiology and Chronic Health Evaluation II; SAPS III, Simplified Acute Physiology Score III; SOFA, Sequential Organ Failure Assessment; Hs, number of secondary injuries, resulting from the sum of the presence of hypotension, hypoxemia, hyperthermia, hypercapnia, hypocapnia, hypoglycaemia, hyponatremia, hypothermia, intracranial hypertension, and clinical evidence of herniation; AUC, area under the receiver operating characteristic curve.

Table S5: Unadjusted odds ratios of prognostic factors for mortality and Unfavourable outcome in the diagnostic group of **traumatic brain injury** in neurocritical patients admitted to intensive care units.

Factors	ICU discharge [a]	Death on ICU [a]	Unadjusted OR (95% CI) for ICU Mortality [b]	p value[c]	Favourable outcome (m-RS score 1, 2, or 3) [a]	Unfavourable outcome (m-RS score 4, 5, or 6) [a]	Unadjusted OR (95% CI) for Unfavourable outcome[b]	p value[c]
Age (years)	(n=162) 54.7 ± 21.5	(n=56) 53 ± 24.2	0.997 (0.983–1.01)	0.625	(n=101) 56.9 ± 21.7	(n=117) 52 ± 22.3	0.99 (0.978–1.002)	0.101
Sex								
Female	48/56 (85.7)	8/56 (14.3)			32/56 (57.1)	24/56 (42.9)		
Male	114/162 (70.4)	48/162 (29.6)	2.526 (1.112–5.741)	0.027	69/162 (42.6)	93/162 (57.4)	1.797 (0.972–3.321)	0.061
Glasgow Coma Scale	(n=162) 12.5 (5–15)	(n=56) 4.5 (3–10)	0.868 (0.812–0.928)	0.000	(n=101) 14 (11–15)	(n=117) 5 (3–9)	0.76 (0.709–0.816)	0.000
APACHE II	(n=162) 12 (8–16)	(n=56) 23.5 (15–27.5)	1.212 (1.145–1.282)	0.000	(n=101) 10 (7–14)	(n=117) 18 (14–24)	1.268 (1.186–1.356)	0.000
SAPS III	(n=159) 45 (37–53)	(n=52) 55.5 (44–68)	1.054 (1.029–1.079)	0.000	(n=99) 42 (34–50)	(n=112) 55 (44.5–63.5)	1.073 (1.047–1.101)	0.000
SOFA	(n=158) 3 (1–7)	(n=51) 9 (6–11)	1.353 (1.224–1.497)	0.000	(n=98) 2 (1–4)	(n=111) 8 (5–10)	1.606 (1.417–1.82)	0.000
Number of Hs								
Zero	60/64 (93.7)	4/64 (6.3)			48/64 (74.6)	16/64 (25.4)		
One	46/51 (90.2)	5/51 (9.8)	1.63 (0.414–6.415)	0.484	33/51 (64.5)	18/51 (35.5)	1.636 (0.731–3.664)	0.231
Two	33/49 (67.3)	16/49 (32.7)	7.273 (2.246–23.553)	0.001	14/49 (28.9)	35/49 (71.1)	7.50 (3.240–17.359)	0.000
Three or more	23/54 (42.6)	31/54 (57.4)	20.217 (6.422–63.651)	0.000	6/54 (11.7)	48/54 (88.3)	24.00 (8.654–66.557)	0.000
Coverage of hospitalization costs								
Private insurance	58/66 (87.9)	8/66 (12.1)			50/66 (75.2)	16/66 (24.8)		
Public insurance	104/152 (68.4)	48/152 (31.6)	3.346 (1.482–7.556)	0.004	51/152 (33.6)	101/152 (66.4)	6.189 (3.212–11.926)	0.000

[a] Categorical variables are described as the absolute number of deaths (percentage of deaths by the total number of cases in the line); age is described as mean ± standard deviation, and the other quantitative variables are described as median (first quartile – third quartile).

[b] Odds ratios and 95% confidence intervals of the univariate binary logistic regression model.

[c] Wald test p value, results < 0.05 indicate statistical significance.

Abbreviations: ICU, intensive care unit; m-RS, modified Rankin scale score; OR, odds ratio; ref., reference; APACHE II, Acute Physiology and Chronic Health Evaluation II; SAPS III, Simplified Acute Physiology Score III; SOFA, Sequential Organ Failure Assessment; Hs, number of secondary injuries, resulting from the sum of the presence of hypotension, hypoxemia, hyperthermia, hypercapnia, hypocapnia, hypoglycaemia, hyponatremia, hypothermia, intracranial hypertension, and clinical evidence of herniation.

Table S6: Adjusted odds ratios of prognostic factors for mortality and Unfavourable outcome in the diagnostic group of **traumatic brain injury** in neurocritical patients admitted to intensive care units.

**Factors**	**Models for ICU mortality**		**Models for Unfavourable outcome (m-RS 4, 5, or 6)**	
**First multivariate model**	**Adjusted OR (95% CI)[b]**	**p value [c]**	**Adjusted OR (95% CI)[b]**	**p value [c]**
Age (years)	1.017 (0.998–1.036)	0.082	1.023 (1.003–1.043)	0.023
Male sex (ref. female)	2.451 (0.951–6.316)	0.063	1.572 (0.671–3.684)	0.298
Glasgow Coma Scale	0.924 (0.844–1.011)	0.085	0.777 (0.708–0.854)	0.000
Number of Hs (ref. zero)				
One	1.553 (0.385–6.266)	0.537	1.354 (0.53–3.463)	0.527
Two	5.564 (1.574–19.675)	0.008	4.471 (1.65–12.119)	0.003
Three or more	16.255 (4.576–57.743)	0.000	10.88 (3.392–34.906)	0.000
Public health insurance coverage (ref. private)	0.961 (0.301–3.068)	0.947	1.146 (0.392–3.351)	0.803
N included in the model [a]	218		218	
AUC (95% CI) [d]	0.819 (0.754–0.884)	0.000	0.877 (0.831–0.923)	0.000
**Second multivariate model**	**Adjusted OR (95% CI)[b]**	**p value [c]**	**Adjusted OR (95% CI)[b]**	**p value [c]**
Male sex (ref. female)	2.922 (1.081–7.896)	0.035	1.58 (0.706–3.535)	0.266
APACHE II score	1.221 (1.148–1.298)	0.000	1.244 (1.161–1.334)	0.000
Public health insurance coverage (ref. private)	0.71 (0.259–1.945)	0.505	2.052 (0.921–4.572)	0.079
N included in the model [a]	218		218	
AUC (95% CI) [d]	0.843 (0.786–0.899)	0.000	0.857 (0.808–0.905)	0.000
**Third multivariate model**	**Adjusted OR (95% CI)[b]**	**p value [c]**	**Adjusted OR (95% CI)[b]**	**p value [c]**
Male sex (ref. female)	2.998 (1.182–7.608)	0.021	1.905 (0.823–4.411)	0.133
SAPS III score	1.061 (1.034–1.088)	0.000	1.093 (1.06–1.128)	0.000
Public health insurance coverage (ref. private)	2.64 (1.098–6.347)	0.030	7.811 (3.538–17.243)	0.000
N included in the model [a]	211		211	
AUC (95% CI) [d]	0.752 (0.679–0.825)	0.000	0.827 (0.772–0.882)	0.000
**Fourth multivariate model**	**Adjusted OR (95% CI)[b]**	**p value [c]**	**Adjusted OR (95% CI)[b]**	**p value [c]**
Age (years)	1.011 (0.992–1.029)	0.264	1.008 (0.99–1.026)	0.409
Male sex (ref. female)	2.164 (0.85–5.51)	0.105	1.259 (0.541–2.929)	0.593
SOFA score	1.364 (1.221–1.524)	0.000	1.561 (1.362–1.789)	0.000
Public health insurance coverage (ref. private)	1.113 (0.371–3.339)	0.849	1.73 (0.639–4.685)	0.281
N included in the model [a]	209		209	
AUC (95% CI) [d]	0.808 (0.742–0.873)	0.000	0.865 (0.815–0.915)	0.000

[a] Number of cases (n) included in the multivariate model. [b] Odds ratios and 95% confidence intervals of the multivariate binary logistic regression model. [c] Wald test p value, results < 0.05 indicate statistical significance.
[d] Area under the receiver operating characteristic curve and 95% confidence interval. Abbreviations: ICU, intensive care unit; m-RS, modified Rankin scale score; OR, odds ratio; 95% CI, 95% confidence interval; ref., reference; AUC, area under the receiver operating characteristic curve; APACHE II, Acute Physiology and Chronic Health Evaluation II; SAPS III, Simplified Acute Physiology Score III; SOFA, Sequential Organ Failure Assessment; Hs, number of secondary injuries, resulting from the sum of the presence of hypotension, hypoxemia, hyperthermia, hypercapnia, hypocapnia, hypoglycaemia, hyponatremia, hypothermia, intracranial hypertension, and clinical evidence of herniation.

Table S7: Unadjusted odds ratios of prognostic factors for mortality and Unfavourable outcome in the diagnostic group of **ischaemic stroke** in neurocritical patients admitted to intensive care units.

Factors	ICU discharge [a]	Death on ICU [a]	Unadjusted OR (95% CI) for ICU Mortality [b]	p value[c]	Favourable outcome (m-RS score 1, 2, or 3) [a]	Unfavourable outcome (m-RS score 4, 5, or 6) [a]	Unadjusted OR (95% CI) for Unfavourable outcome[b]	p value[c]
Age (years)	(n=187) 69.2 ± 17	(n=24) 69.7 ± 15.8	1.002 (0.976–1.027)	0.906	(n=126) 68.8 ± 16.8	(n=82) 69.8 ± 17.1	1.003 (0.987–1.02)	0.696
Sex								
Female	92/104 (88.5)	12/104 (11.5)			58/103 (56.3)	45/103 (43.7)		
Male	95/107 (88.8)	12/107 (11.2)	0.968 (0.414–2.266)	0.941	68/105 (64.8)	37/105 (35.2)	0.701 (0.401–1.226)	0.213
Glasgow Coma Scale	(n=187) 15 (13–15)	(n=24) 10.5 (5–13.5)	0.775 (0.699–0.86)	0.000	(n=126) 15 (14–15)	(n=82) 11 (8–14)	0.633 (0.541–0.74)	0.000
APACHE II	(n=187) 10 (7–14)	(n=24) 18.5 (14.5–21.5)	1.206 (1.114–1.307)	0.000	(n=126) 9 (6–12)	(n=82) 15 (10–19)	1.182 (1.116–1.253)	0.000
SAPS III	(n=180) 52 (42–57)	(n=21) 59 (44–67)	1.049 (1.014–1.085)	0.006	(n=122) 48 (41–54)	(n=176) 56 (46–62.5)	1.056 (1.028–1.085)	0.000
SOFA	(n=175) 1 (0–4)	(n=21) 6 (3–9)	1.239 (1.104–1.391)	0.000	(n=118) 1 (0–3)	(n=75) 5 (2–7)	1.332 (1.195–1.486)	0.000
Number of Hs								
Zero	108/109 (99.1)	1/109 (0.9)			82/106 (77.4)	24/106 (22.6)		
One	47/55 (85.5)	8/55 (14.5)	18.383 (2.236–151.155)	0.007	29/55 (52.7)	26/55 (47.3)	3.063 (1.524–6.156)	0.002
Two	23/30 (76.7)	7/30 (23.3)	32.87 (3.855–280.237)	0.001	12/30 (39.4)	18/30 (60.6)	5.125 (2.168–12.117)	0.000
Three or more	9/17 (52.9)	8/17 (47.1)	96 (10.774–855.42)	0.000	3/17 (17.6)	14/17 (82.4)	15.944 (4.228–60.125)	0.000
Coverage of hospitalization costs								
Private insurance	157/167 (94)	10/167 (6)			114/164 (69.5)	50/164 (30.5)		
Public insurance	30/44 (68.2)	14/44 (31.8)	7.327 (2.977–18.031)	0.000	32/44 (27.3)	32/44 (72.7)	6.08 (2.895–12.77)	0.000

[a] Categorical variables are described as the absolute number of deaths (percentage of deaths by the total number of cases in the line); age is described as mean ± standard deviation, and the other quantitative variables are described as median (first quartile – third quartile).
[b] Odds ratio and 95% confidence interval of the univariate binary logistic regression model.
[c] Wald test p value, results < 0.05 indicate statistical significance.
Abbreviations: ICU, intensive care unit; m-RS, modified Rankin scale score; OR, odds ratio; ref., reference; APACHE II, Acute Physiology and Chronic Health Evaluation II; SAPS III, Simplified Acute Physiology Score III; SOFA, Sequential Organ Failure Assessment; Hs, number of secondary injuries, resulting from the sum of the presence of hypotension, hypoxemia, hyperthermia, hypercapnia, hypocapnia, hypoglycaemia, hyponatremia, hypothermia, intracranial hypertension, and clinical evidence of herniation.

Table S8: Adjusted odds ratios of prognostic factors for mortality and Unfavourable outcome in the diagnostic group of **ischaemic stroke** in neurocritical patients admitted to intensive care units.

**Factors**	**Models for ICU mortality**		**Models for Unfavourable outcome (m-RS 4, 5, or 6)**	
**First multivariate model**	**Adjusted OR (95% CI)** [b]	**p value** [c]	**Adjusted OR (95% CI)** [b]	**p value** [c]
Age (years)	1.033 (0.994–1.074)	0.102	1.007 (0.985–1.03)	0.517
Male sex (ref. female)	1.029 (0.366–2.891)	0.956	0.61 (0.301–1.234)	0.169
Glasgow Coma Scale	0.868 (0.768–0.982)	0.024	0.678 (0.573–0.803)	0.000
Number of Hs (ref. zero)				
One	8.751 (0.993–77.144)	0.051	1.576 (0.678–3.663)	0.291
Two	17.361 (1.901–158.572)	0.011	2.783 (1.005–7.703)	0.049
Three or more	54.814 (5.672–529.691)	0.001	8.944 (2.038–39.258)	0.004
Public health insurance coverage (ref. private)	3.604 (1.041–12.474)	0.043	1.997 (0.73–5.463)	0.178
N included in the model [a]	211		208	
AUC (95% CI) [d]	0.896 (0.841–0.951)	0.000	0.858 (0.804–0.913)	0.000
**Second multivariate model**	**Adjusted OR (95% CI)** [b]	**p value** [c]	**Adjusted OR (95% CI)** [b]	**p value** [c]
Male sex (ref. female)	0.921 (0.348–2.438)	0.868	0.58 (0.3–1.124)	0.107
APACHE II score	1.195 (1.098–1.3)	0.000	1.166 (1.1–1.236)	0.000
Public health insurance coverage (ref. private)	5.878 (2.22–15.566)	0.000	5.42 (2.384–12.323)	0.000
N included in the model [a]	211		208	
AUC (95% CI) [d]	0.857 (0.786–0.928)	0.000	0.804 (0.744–0.864)	0.000
**Third multivariate model**	**Adjusted OR (95% CI)** [b]	**p value** [c]	**Adjusted OR (95% CI)** [b]	**p value** [c]
Male sex (ref. female)	1.049 (0.39–2.819)	0.924	0.594 (0.31–1.141)	0.118
SAPS III score	1.044 (1.01–1.079)	0.011	1.06 (1.029–1.091)	0.000
Public health insurance coverage (ref. private)	6.191 (2.315–16.557)	0.000	7.424 (3.088–17.849)	0.000
N included in the model [a]	201		198	
AUC (95% CI) [d]	0.767 (0.655–0.88)	0.000	0.756 (0.687–0.825)	0.000
**Fourth multivariate model**	**Adjusted OR (95% CI)** [b]	**p value** [c]	**Adjusted OR (95% CI)** [b]	**p value** [c]
Age (years)	1.022 (0.987–1.057)	0.222	1.013 (0.991–1.035)	0.241
Male sex (ref. female)	1.257 (0.456–3.466)	0.659	0.701 (0.359–1.369)	0.298
SOFA score	1.204 (1.064–1.363)	0.003	1.262 (1.129–1.41)	0.000
Public health insurance coverage (ref. private)	5.916 (1.943–18.012)	0.002	4.511 (1.839–11.069)	0.001
N included in the model [a]	197		193	
AUC (95% CI) [d]	0.827 (0.751–0.903)	0.000	0.806 (0.744–0.868)	0.000

[a] Number of cases (n) included in the multivariate model. [b] Odds ratios and 95% confidence intervals of the multivariate binary logistic regression model. [c] Wald test p value, results < 0.05 indicate statistical significance.

[d]Area under the receiver operating characteristic curve and 95% confidence interval. Abbreviations: ICU, intensive care unit; m-RS, modified Rankin scale score; OR, odds ratio; 95% CI, 95% confidence interval; ref., reference; AUC, area under the receiver operating characteristic curve; APACHE II, Acute Physiology and Chronic Health Evaluation II; SAPS III, Simplified Acute Physiology Score III; SOFA, Sequential Organ Failure Assessment; Hs, number of secondary injuries, resulting from the sum of the presence of hypotension, hypoxemia, hyperthermia, hypercapnia, hypocapnia, hypoglycaemia, hyponatremia, hypothermia, intracranial hypertension, and clinical evidence of herniation

Table S9: Unadjusted odds ratios of prognostic factors for mortality and Unfavourable outcome in the diagnostic group of encephalopathy **in neurocritical patients** admitted to intensive care units.

**Factors**	**ICU discharge** [a]	**Death on ICU** [a]	**Unadjusted OR (95% CI) for ICU Mortality** [b]	**p value**[c]	**Favourable outcome (m-RS score 1, 2, or 3)** [a]	**Unfavourable outcome (m-RS score 4, 5, or 6)** [a]	**Unadjusted OR (95% CI) for Unfavourable outcome**[b]	**p value**[c]
Age (years)	(n=114) 64 ± 20.2	(n=41) 70.2 ± 19.4	1.016 (0.997–1.036)	0.093	(n=66) 63.2 ± 19.9	(n=89) 67.5 ± 20.3	1.011 (0.995–1.027)	0.195
Sex								
Female	66/83 (79.5)	17/83 (20.5)			40/83 (48.2)	43/83 (51.8)		
Male	48/72 (66.7)	24/72 (33.3)	1.941 (0.941–4.004)	0.073	26/72 (36.1)	46/72 (63.9)	1.646 (0.863–3.138)	0.130
Glasgow Coma Scale	(n=114) 13 (10–14)	(n=41) 10 (3–14)	0.872 (0.8–0.95)	0.002	(n=66) 14 (12–14)	(n=89) 11 (8–14)	0.864 (0.789–0.947)	0.002
APACHE II	(n=114) 13.5 (9–20)	(n=41) 27 (20–33)	1.135 (1.083–1.188)	0.000	(n=66) 11 (8–15)	(n=89) 22 (14–28)	1.136 (1.083–1.191)	0.000
SAPS III	(n=113) 54 (44–63)	(n=41) 69 (59–78)	1.074 (1.042–1.106)	0.000	(n=65) 50 (41–57)	(n=89) 64 (55–75)	1.083 (1.051–1.116)	0.000
SOFA	(n=114) 3 (1–7)	(n=41) 9 (5–11)	1.308 (1.175–1.456)	0.000	(n=66) 2 (1–6)	(n=89) 7 (4–10)	1.288 (1.163–1.427)	0.000
Number of Hs								
Zero	44/47 (93.6)	3/47 (6.4)			30/47 (63.8)	17/47 (36.2)		
One	32/39 (82.1)	7/39 (17.9)	3.208 (0.77–13.368)	0.109	18/39 (46.2)	21/39 (53.8)	2.059 (0.866–4.896)	0.102
Two	21/32 (65.6)	11/32 (34.4)	7.683 (1.936–30.488)	0.004	12/32 (37.5)	20/32 (62.5)	2.941 (1.16–7.46)	0.023
Three or more	17/37 (45.9)	20/37 (54.1)	17.255 (4.535–65.657)	0.000	6/37 (16.2)	31/37 (83.8)	9.118 (3.167–26.25)	0.000
Coverage of hospitalization costs								
Private insurance	70/89 (78.7)	19/89 (21.3)			45/89 (50.6)	44/89 (49.4)		
Public insurance	44/66 (66.7)	22/66 (33.3)	1.842 (0.896–3.786)	0.096	21/66 (31.8)	45/66 (68.2)	2.192 (1.128–4.257)	0.021

[a] Categorical variables are described as the absolute number of deaths (percentage of deaths by the total number of cases in the line); age is described as mean ± standard deviation, and the other quantitative variables are described as median (first quartile – third quartile).

[b] Odds ratio and 95% confidence interval of the univariate binary logistic regression model.

[c] Wald test p value, results < 0.05 indicate statistical significance.

Abbreviations: ICU, intensive care unit; m-RS, modified Rankin scale score; OR, odds ratio; ref., reference; APACHE II, Acute Physiology and Chronic Health Evaluation II; SAPS III, Simplified Acute Physiology Score III; SOFA, Sequential Organ Failure Assessment; Hs, number of secondary injuries, resulting from the sum of the presence of hypotension, hypoxemia, hyperthermia, hypercapnia, hypocapnia, hypoglycaemia, hyponatremia, hypothermia, intracranial hypertension, and clinical evidence of herniation.

Table S10: Adjusted odds ratios of prognostic factors for mortality and Unfavourable outcome in the diagnostic group of **encephalopathy** in neurocritical patients admitted to intensive care units.

**Factors**	**Models for ICU mortality**		**Models for Unfavourable outcome (m-RS 4, 5, or 6)**	
**First multivariate model**	**Adjusted OR (95% CI)** [b]	**p value** [c]	**Adjusted OR (95% CI)** [b]	**p value** [c]
Age (years)	1.035 (1.009–1.062)	0.008	1.026 (1.005–1.047)	0.013
Male sex (ref. female)	2.827 (1.169–6.838)	0.021	1.766 (0.847–3.68)	0.129
Glasgow Coma Scale	0.915 (0.824–1.017)	0.099	0.905 (0.816–1.005)	0.062
Number of Hs (ref. zero Hs)				
One	2.922 (0.661–12.918)	0.157	1.734 (0.69–4.359)	0.242
Two	7.119 (1.614–31.397)	0.010	2.268 (0.822–6.258)	0.114
Three or more	19.792 (4.447–88.1)	0.000	7.778 (2.444–24.75)	0.001
Public health insurance coverage (ref. private)	1.184 (0.446–3.141)	0.734	1.937 (0.852–4.403)	0.115
N included in the model [a]	155		155	
AUC (95% CI) [d]	0.822 (0.751–0.894)	0.000	0.772 (0.697–0.848)	0.000
**Second multivariate model**	**Adjusted OR (95% CI)** [b]	**p value** [c]	**Adjusted OR (95% CI)** [b]	**p value** [c]
Male sex (ref. female)	1.872 (0.803–4.367)	0.147	1.436 (0.683–3.018)	0.340
APACHE II score	1.133 (1.081–1.187)	0.000	1.134 (1.081–1.19)	0.000
Public health insurance coverage (ref. private)	1.364 (0.586–3.178)	0.471	2.015 (0.94–4.317)	0.072
N included in the model [a]	155		155	
AUC (95% CI) [d]	0.816 (0.741–0.891)	0.000	0.799 (0.73–0.869)	0.000
**Third multivariate model**	**Adjusted OR (95% CI)** [b]	**p value** [c]	**Adjusted OR (95% CI)** [b]	**p value** [c]
Male sex (ref. female)	1.809 (0.808–4.052)	0.149	1.462 (0.692–3.089)	0.319
SAPS III score	1.072 (1.04–1.105)	0.000	1.081 (1.049–1.115)	0.000
Public health insurance coverage (ref. private)	1.262 (0.559–2.85)	0.575	1.797 (0.836–3.864)	0.134
N included in the model [a]	154		154	
AUC (95% CI) [d]	0.776 (0.697–0.855)	0.000	0.794 (0.724–0.865)	0.000
**Fourth multivariate model**	**Adjusted OR (95% CI)** [b]	**p value** [c]	**Adjusted OR (95% CI)** [b]	**p value** [c]
Age (years)	1.025 (1–1.05)	0.053	1.015 (0.995–1.035)	0.137
Male sex (ref. female)	2.011 (0.878–4.606)	0.099	1.513 (0.735–3.116)	0.261
SOFA score	1.297 (1.156–1.455)	0.000	1.261 (1.135–1.401)	0.000
Public health insurance coverage (ref. private)	1.433 (0.561–3.662)	0.452	1.881 (0.841–4.207)	0.124
N included in the model [a]	155		155	
AUC (95% CI) [d]	0.796 (0.716–0.876)	0.000	0.765 (0.69–0.841)	0.000

[a] Number of cases (n) included in the multivariate model. [b] Odds ratios and 95% confidence intervals of the multivariate binary logistic regression model. [c] Wald test p value, results < 0.05 indicate statistical significance.
[d] Area under the receiver operating characteristic curve and 95% confidence interval. Abbreviations: ICU, intensive care unit; m-RS, modified Rankin scale score; OR, odds ratio; 95% CI, 95% confidence interval; ref., reference; AUC, area under the receiver operating characteristic curve; APACHE II, Acute Physiology and Chronic Health Evaluation II; SAPS III, Simplified Acute Physiology Score III; SOFA, Sequential Organ Failure Assessment; Hs, number of secondary injuries, resulting from the sum of the presence of hypotension, hypoxemia, hyperthermia, hypercapnia, hypocapnia, hypoglycaemia, hyponatremia, hypothermia, intracranial hypertension, and clinical evidence of herniation.

Table S11: Unadjusted odds ratios of prognostic factors for mortality and Unfavourable outcome in the diagnostic group of **seizures** in neurocritical patients admitted to intensive care units.

**Factors**	**ICU discharge** [a]	**Death on ICU** [a]	**Unadjusted OR (95% CI) for ICU Mortality** [b]	**p value**[c]	**Favourable outcome (m-RS score 1, 2, or 3)** [a]	**Unfavourable outcome (m-RS score 4, 5, or 6)** [a]	**Unadjusted OR (95% CI) for Unfavourable outcome**[b]	**p value**[c]
Age (years)	(n=87) 58.8 ± 23.1	(n=4) 56.5 ± 11.4	0.996 (0.953–1.04)	0.841	(n=64) 56.3 ± 23.4	(n=25) 63.1 ± 19.9	1.014 (0.992–1.036)	0.204
Sex								
Female	42/45 (93.3)	3/45 (6.7)			31/43 (72.1)	12/43 (27.9)		
Male	45/46 (97.8)	1/46 (2.2)	0.311 (0.031–3.109)	0.320	33/46 (71.7)	13/46 (28.3)	1.018 (0.403–2.567)	0.970
Glasgow Coma Scale	(n=87) 14 (11–15)	(n=4) 11 (7.5–14)	0.913 (0.735–1.134)	0.411	(n=64) 15 (14–15)	(n=25) 9 (6–14)	0.781 (0.688–0.886)	0.000
APACHE II	(n=87) 10 (5–15)	(n=4) 15.5 (10.5–18)	1.053 (0.936–1.185)	0.392	(n=64) 8 (4–12)	(n=25) 16 (12–19)	1.15 (1.067–1.239)	0.000
SAPS III	(n=86) 41 (33–54)	(n=4) 40 (37.5–53)	1.007 (0.938–1.081)	0.853	(n=64) 39 (31–48.5)	(n=24) 54 (41.5–65)	1.076 (1.034–1.12)	0.000
SOFA	(n=81) 1 (0–4)	(n=4) 6 (3–7.5)	1.399 (0.997–1.964)	0.052	(n=59) 1 (0–3)	(n=24) 4 (3–6.5)	1.639 (1.289–2.084)	0.000
Number of Hs								
Zero	42/44 (95.5)	2/44 (4.5)			32/42 (76.2)	10/42 (23.8)		
One	23/24 (95.8)	1/24 (4.2)	0.913 (0.078–10.62)	0.942	20/24 (83.3)	4/24 (16.7)	0.64 (0.177–2.318)	0.497
Two	13/14 (92.9)	1/14 (7.1)	1.615 (0.135–19.285)	0.705	7/14 (50)	7/14 (50)	3.2 (0.903–11.345)	0.072
Three or more	9/9 (100)	0/9 (0)	N/A	N/A	5/9 (55.6)	4/9 (44.4)	2.56 (0.574–11.408)	0.218
Coverage of hospitalization costs								
Private insurance	78/79 (98.7)	1/79 (1.3)			59/77 (76.6)	18/77 (23.4)		
Public insurance	9/12 (75)	3/12 (25)	0.913 (0.078–10.62)	0.942	5/12 (41.7)	7/12 (58.3)	4.589 (1.298–16.229)	0.018

[a] Categorical variables are described as the absolute number of deaths (percentage of deaths by the total number of cases in the line); age is described as mean ± standard deviation, and the other quantitative variables are described as median (first quartile – third quartile).
[b] Odds ratios and 95% confidence intervals of the univariate binary logistic regression model.
[c] Wald test p value, results < 0.05 indicate statistical significance.
Abbreviations: ICU, intensive care unit; m-RS, modified Rankin scale score; OR, odds ratio; ref., reference; APACHE II, Acute Physiology and Chronic Health Evaluation II; SAPS III, Simplified Acute Physiology Score III; SOFA, Sequential Organ Failure Assessment; Hs, number of secondary injuries, resulting from the sum of the presence of hypotension, hypoxemia, hyperthermia, hypercapnia, hypocapnia, hypoglycaemia, hyponatremia, hypothermia, intracranial hypertension, and clinical evidence of herniation; N/A, not analysed.

Table S12: Adjusted odds ratios of prognostic factors for mortality and Unfavourable outcome in the diagnostic group of **seizures** in neurocritical patients admitted to intensive care units.

Factors	Models for ICU mortality		Models for Unfavourable outcome (m-RS 4, 5, or 6)	
**First multivariate model**	**Adjusted OR (95% CI)** [b]	**p value** [c]	**Adjusted OR (95% CI)** [b]	**p value** [c]
Age (years)	N/A	N/A	1.025 (0.996–1.055)	0.089
Male sex (ref. female)	N/A	N/A	0.754 (0.241–2.354)	0.627
Glasgow Coma Scale	N/A	N/A	0.778 (0.672–0.9)	0.001
Number of Hs (ref. zero Hs)		N/A		
One	N/A	N/A	0.548 (0.133–2.266)	0.406
Two	N/A	N/A	1.478 (0.308–7.101)	0.625
Three or more	N/A	N/A	0.776 (0.124–4.873)	0.787
Public health insurance coverage (ref. private)	N/A	N/A	5.028 (0.965–26.187)	0.055
N included in the model [a]	N/A	N/A	89	
AUC (95% CI) [d]	N/A	N/A	0.888 (0.819–0.956)	0.000
**Second multivariate model**	**Adjusted OR (95% CI)** [b]	**p value** [c]	**Adjusted OR (95% CI)** [b]	**p value** [c]
Male sex (ref. female)	0.042 (0.002–1.128)	0.059	0.723 (0.243–2.151)	0.559
APACHE II score	1.062 (0.897–1.257)	0.486	1.149 (1.063–1.241)	0.000
Public health insurance coverage (ref. private)	91.444 (4.383–1907.891)	0.004	4.532 (1.081–19.006)	0.039
N included in the model [a]	91		89	
AUC (95% CI) [d]	0.943 (0.867–1)	0.003	0.835 (0.748–0.922)	0.000
**Third multivariate model**	**Adjusted OR (95% CI)** [b]	**p value** [c]	**Adjusted OR (95% CI)** [b]	**p value** [c]
Male sex (ref. female)	0.06 (0.003–1.295)	0.073	0.911 (0.306–2.706)	0.866
SAPS III score	0.985 (0.896–1.082)	0.750	1.078 (1.033–1.124)	0.000
Public health insurance coverage (ref. private)	102.753 (4.714–2239.75)	0.003	4.193 (0.971–18.095)	0.055
N included in the model [a]	90		88	
AUC (95% CI) [d]	0.863 (0.644–1)	0.014	0.823 (0.724–0.921)	0.000
**Fourth multivariate model**	**Adjusted OR (95% CI)** [b]	**p value** [c]	**Adjusted OR (95% CI)** [b]	**p value** [c]
Age (years)	1.008 (0.937–1.085)	0.822	1.018 (0.99–1.047)	0.209
Male sex (ref. female)	0.064 (0.002–1.795)	0.106	0.713 (0.221–2.305)	0.572
SOFA score	1.318 (0.838–2.073)	0.233	1.595 (1.25–2.036)	0.000
Public health insurance coverage (ref. private)	71.58 (2.584–1983.206)	0.012	3.074 (0.613–15.423)	0.172
N included in the model [a]	85		83	
AUC (95% CI) [d]	0.957 (0.89–1)	0.002	0.846 (0.765–0.927)	0.000

[a] Number of cases (n) included in the multivariate model. [b] Odds ratios and 95% confidence intervals of the multivariate binary logistic regression model. [c] Wald test p value, results < 0.05 indicate statistical significance.
[d] Area under the receiver operating characteristic curve and 95% confidence interval. Abbreviations: N/A, not analysed; ICU, intensive care unit; m-RS, modified Rankin scale score; OR, odds ratio; 95% CI, 95% confidence interval; ref., reference; AUC, area under the receiver operating characteristic curve; APACHE II, Acute Physiology and Chronic Health Evaluation II; SAPS III, Simplified Acute Physiology Score III; SOFA, Sequential Organ Failure Assessment; Hs, number of secondary injuries, resulting from the sum of the presence of hypotension, hypoxemia, hyperthermia, hypercapnia, hypocapnia, hypoglycaemia, hyponatremia, hypothermia, intracranial hypertension, and clinical evidence of herniation.

Table S13: Unadjusted odds ratios of prognostic factors for mortality and Unfavourable outcome in the diagnostic group of **intracerebral haemorrhage** in neurocritical patients admitted to intensive care units.

**Factors**	**ICU discharge** [a]	**Death on ICU** [a]	**Unadjusted OR (95% CI) for ICU Mortality** [b]	**p value**[c]	**Favourable outcome (m-RS score 1, 2, or 3)** [a]	**Unfavourable outcome (m-RS score 4, 5, or 6)** [a]	**Unadjusted OR (95% CI) for Unfavourable outcome**[b]	**p value**[c]
Age (years)	(n=55) 63.2 ± 13.5	(n=22) 59 ± 19.2	0.982 (0.951–1.015)	0.280	(n=24) 65.4 ± 13.5	(n=52) 60.2 ± 16	0.977 (0.944–1.01)	0.174
Sex								
Female	27/40 (67.5)	13/40 (32.5)			7/39 (17.9)	32/39 (82.1)		
Male	28/37 (75.7)	9/37 (24.3)	0.668 (0.245–1.816)	0.429	17/37 (45.9)	20/37 (54.1)	0.257 (0.091–0.73)	0.011
Glasgow Coma Scale	(n=55) 11 (4–14)	(n=22) 7 (4–13)	0.916 (0.822–1.021)	0.115	(n=24) 14 (13–15)	(n=52) 7 (3.5–12)	0.732 (0.623–0.86)	0.000
APACHE II	(n=55) 14 (9–19)	(n=22) 18.5 (14–23)	1.086 (1.007–1.171)	0.031	(n=24) 10.5 (7.5–14.5)	(n=52) 18 (14–23)	1.222 (1.097–1.362)	0.000
SAPS III	(n=51) 52 (44–60)	(n=21) 63 (47–69)	1.031 (0.996–1.067)	0.084	(n=22) 42.5 (34–52)	(n=49) 60 (51–69)	1.11 (1.051–1.172)	0.000
SOFA	(n=50) 4 (2–6)	(n=20) 7 (4.5–10)	1.27 (1.071–1.506)	0.006	(n=22) 2 (1–4)	(n=47) 6 (4–8)	1.71 (1.278–2.287)	0.000
Number of Hs								
Zero	24/30 (80)	6/30 (20)			14/29 (48.3)	15/29 (51.7)		
One	16/20 (80)	4/20 (20)	1 (0.243–4.114)	1.000	8/20 (40)	12/20 (60)	1.4 (0.442–4.437)	0.568
Two	9/13 (69.2)	4/13 (30.8)	1.778 (0.405–7.802)	0.446	2/13 (15.4)	11/13 (84.6)	5.133 (0.963–27.363)	0.055
Three or more	6/14 (42.9)	8/14 (57.1)	5.333 (1.334–21.325)	0.018	0/14 (0)	14/14 (100)	All with outcome	
Coverage of hospitalization costs								
Private insurance	19/25 (76)	6/25 (24)			11/24 (45.8)	13/24 (54.2)		
Public insurance	36/52 (69.2)	16/52 (30.8)	1.407 (0.473–4.188)	0.539	13/52 (25)	39/52 (75)	2.538 (0.916–7.034)	0.073

[a] Categorical variables are described as the absolute number of deaths (percentage of deaths by the total number of cases in the line); age is described as mean ± standard deviation, and the other quantitative variables are described as median (first quartile – third quartile).

[b] Odds ratios and 95% confidence intervals of the univariate binary logistic regression model.

[c] Wald test p value, results < 0.05 indicate statistical significance.

Abbreviations: ICU, intensive care unit; m-RS, modified Rankin scale score; OR, odds ratio; ref., reference; APACHE II, Acute Physiology and Chronic Health Evaluation II; SAPS III, Simplified Acute Physiology Score III; SOFA, Sequential Organ Failure Assessment; Hs, number of secondary injuries, resulting from the sum of the presence of hypotension, hypoxemia, hyperthermia, hypercapnia, hypocapnia, hypoglycaemia, hyponatremia, hypothermia, intracranial hypertension, and clinical evidence of herniation.

Table S14: Adjusted odds ratios of prognostic factors for mortality and Unfavourable outcome in the diagnostic group of the **intracerebral haemorrhage** in neurocritical patients admitted to intensive care units.

**Factors**	**Models for ICU mortality**		**Models for Unfavourable outcome (m-RS 4, 5, or 6)**	
**First multivariate model**	**Adjusted OR (95% CI)** [b]	**p value** [c]	**Adjusted OR (95% CI)** [b]	**p value** [c]
Age (years)	0.983 (0.948–1.019)	0.353	0.971 (0.919–1.027)	0.307
Male sex (ref. female)	0.759 (0.252–2.288)	0.625	0.156 (0.035–0.697)	0.015
Glasgow Coma Scale	0.978 (0.861–1.11)	0.732	0.731 (0.602–0.887)	0.002
Number of Hs (ref. zero Hs)				
One	1.03 (0.241–4.406)	0.968	2.94 (0.625–13.836)	0.172
Two	1.693 (0.363–7.902)	0.503	5.087 (0.557–46.442)	0.149
Three or more	4.89 (1.113–21.477)	0.036	All with outcome	
Public health insurance coverage (ref. private)	1.208 (0.35–4.169)	0.765	1.301 (0.257–6.589)	0.751
N included in the model [a]	77		76	
AUC (95% CI) [d]	0.693 (0.563–0.824)	0.008	0.903 (0.832–0.975)	0.000
**Second multivariate model**	**Adjusted OR (95% CI)** [b]	**p value** [c]	**Adjusted OR (95% CI)** [b]	**p value** [c]
Male sex (ref. female)	0.812 (0.285–2.317)	0.697	0.302 (0.09–1.009)	0.052
APACHE II score	1.087 (1.005–1.175)	0.036	1.213 (1.088–1.353)	0.001
Public health insurance coverage (ref. private)	1.578 (0.5–4.975)	0.437	3.433 (0.945–12.467)	0.061
N included in the model [a]	77		76	
AUC (95% CI) [d]	0.662 (0.534–0.79)	0.027	0.853 (0.77–0.936)	0.000
**Third multivariate model**	**Adjusted OR (95% CI)** [b]	**p value** [c]	**Adjusted OR (95% CI)** [b]	**p value** [c]
Male sex (ref. female)	0.708 (0.241–2.078)	0.529	0.377 (0.103–1.381)	0.141
SAPS III score	1.029 (0.993–1.066)	0.113	1.109 (1.048–1.173)	0.000
Public health insurance coverage (ref. private)	1.403 (0.447–4.406)	0.562	3.635 (0.96–13.766)	0.058
N included in the model [a]	72		71	
AUC (95% CI) [d]	0.648 (0.504–0.791)	0.050	0.865 (0.774–0.955)	0.000
**Fourth multivariate model**	**Adjusted OR (95% CI)** [b]	**p value** [c]	**Adjusted OR (95% CI)** [b]	**p value** [c]
Age (years)	0.979 (0.943–1.016)	0.258	0.992 (0.944–1.042)	0.740
Male sex (ref. female)	0.585 (0.184–1.858)	0.363	0.272 (0.073–1.01)	0.052
SOFA score	1.258 (1.058–1.497)	0.009	1.619 (1.198–2.188)	0.002
Public health insurance coverage (ref. private)	0.807 (0.22–2.96)	0.746	2.378 (0.52–10.876)	0.264
N included in the model [a]	70		69	
AUC (95% CI) [d]	0.725 (0.584–0.866)	0.003	0.87 (0.773–0.968)	0.000

[a] Number of cases (n) included in the multivariate model. [b] Odds ratios and 95% confidence intervals of the multivariate binary logistic regression model. [c] Wald test p value, results < 0.05 indicate statistical significance.
[d] Area under the receiver operating characteristic curve and 95% confidence interval. Abbreviations: ICU, intensive care unit; m-RS, modified Rankin scale score; OR, odds ratio; 95% CI, 95% confidence interval; ref., reference; AUC, area under the receiver operating characteristic curve; APACHE II, Acute Physiology and Chronic Health Evaluation II; SAPS III, Simplified Acute Physiology Score III; SOFA, Sequential Organ Failure Assessment; Hs, number of secondary injuries, resulting from the sum of the presence of hypotension, hypoxemia, hyperthermia, hypercapnia, hypocapnia, hypoglycaemia, hyponatremia, hypothermia, intracranial hypertension, and clinical evidence of herniation.

Table S15: Unadjusted odds ratios of prognostic factors for mortality and Unfavourable outcome in the diagnostic group of subarachnoid haemorrhage in neurocritical patients admitted to intensive care units.

Factors	ICU discharge [a]	Death on ICU [a]	Unadjusted OR (95% CI) for ICU Mortality [b]	p value[c]	Favourable outcome (m-RS score 1, 2, or 3) [a]	Unfavourable outcome (m-RS score 4, 5, or 6) [a]	Unadjusted OR (95% CI) for Unfavourable outcome[b]	p value[c]
Age (years)	(n=46) 55.2 ± 11.4	(n=24) 62.2 ± 17.9	1.037 (0.999–1.076)	0.059	(n=26) 54.8 ± 10	(n=44) 59.3 ± 16.1	1.024 (0.987–1.062)	0.201
Sex								
Female	32/48 (66.7)	16/48 (33.3)			20/48 (41.7)	28/48 (58.3)		
Male	14/22 (63.6)	8/22 (36.4)	1.143 (0.398–3.285)	0.804	6/22 (27.3)	16/22 (72.7)	1.905 (0.634–5.721)	0.251
Glasgow Coma Scale	(n=46) 14 (8–15)	(n=24) 3 (3–9)	0.784 (0.692–0.888)	0.000	(n=26) 14 (11–15)	7 (3–12)	0.788 (0.691–0.898)	0.000
APACHE II	(n=46) 10.5 (6–16)	(n=24) 20 (17–30)	1.184 (1.085–1.293)	0.000	(n=26) 7 (5–11)	(n=44) 18 (13.5–23)	1.267 (1.129–1.421)	0.000
SAPS III	(n=44) 46 (36.5–48.5)	(n=21) 60 (52–76)	1.062 (1.023–1.102)	0.002	(n=25) 42 (34–48)	(n=40) 54 (45.5–70.5)	1.083 (1.03–1.139)	0.002
SOFA	(n=43) 3 (1–5)	(n=21) 7 (5–9)	1.367 (1.15–1.625)	0.000	(n=25) 2 (0–3)	(n=39) 6 (3–9)	1.412 (1.156–1.724)	0.001
Number of Hs								
Zero	21/24 (87.5)	3/24 (12.5)			13/24 (54.2)	11/24 (45.8)		
One	14/20 (70)	6/20 (30)	3 (0.642–14.023)	0.163	6/20 (30)	14/20 (70)	2.758 (0.791–9.613)	0.111
Two	7/14 (50)	7/14 (50)	7 (1.413–34.682)	0.017	4/14 (28.6)	10/14 (71.4)	2.955 (0.721–12.107)	0.132
Three or more	4/12 (33.3)	8/12 (66.7)	14 (2.547–76.95)	0.002	3/12 (25)	9/12 (75)	3.545 (0.765–16.433)	0.106
Coverage of hospitalization costs								
Private insurance	7/12 (58.3)	5/12 (41.7)			6/12 (50)	6/12 (50)		
Public insurance	39/58 (67.2)	19/58 (32.8)	0.682 (0.191–2.433)	0.555	20/58 (34.5)	38/58 (65.5)	1.9 (0.542–6.661)	0.316

[a] Categorical variables are described as the absolute number of deaths (percentage of deaths by the total number of cases in the line); age is described as mean ± standard deviation, and the other quantitative variables are described as median (first quartile – third quartile).

[b] Odds ratios and 95% confidence intervals of the univariate binary logistic regression model.

[c] Wald test p value, results < 0.05 indicate statistical significance.

Abbreviations: ICU, intensive care unit; m-RS, modified Rankin scale score; OR, odds ratio; ref., reference; APACHE II, Acute Physiology and Chronic Health Evaluation II; SAPS III, Simplified Acute Physiology Score III; SOFA, Sequential Organ Failure Assessment; Hs, number of secondary injuries, resulting from the sum of the presence of hypotension, hypoxemia, hyperthermia, hypercapnia, hypocapnia, hypoglycaemia, hyponatremia, hypothermia, intracranial hypertension, and clinical evidence of herniation.

Table S16: Adjusted odds ratios of prognostic factors for mortality and Unfavourable outcome in the diagnostic group of subarachnoid haemorrhage in neurocritical patients admitted to intensive care units.

**Factors**	**Models for ICU mortality**		**Models for Unfavourable outcome (m-RS 4, 5, or 6)**	
**First multivariate model**	**Adjusted OR (95% CI)** [b]	**p value** [c]	**Adjusted OR (95% CI)** [b]	**p value** [c]
Age (years)	1.025 (0.976–1.076)	0.323	1.025 (0.978–1.075)	0.307
Male sex (ref. female)	0.94 (0.236–3.742)	0.930	2.334 (0.624–8.728)	0.208
Glasgow Coma Scale	0.805 (0.688–0.941)	0.006	0.748 (0.62–0.904)	0.003
Number of Hs (ref. zero Hs)				
One	1.978 (0.301–13.002)	0.478	0.968 (0.215–4.363)	0.967
Two	6.252 (0.969–40.359)	0.054	1.724 (0.32–9.281)	0.526
Three or more	4.183 (0.464–37.713)	0.202	0.242 (0.02–2.936)	0.265
Public health insurance coverage (ref. private)	0.408 (0.056–2.949)	0.374	3.808 (0.612–23.703)	0.152
N included in the model [a]	70		70	
AUC (95% CI) [d]	0.844 (0.744–0.944)	0.000	0.821 (0.715–0.928)	0.000
**Second multivariate model**	**Adjusted OR (95% CI)** [b]	**p value** [c]	**Adjusted OR (95% CI)** [b]	**p value** [c]
Male sex (ref. female)	1.066 (0.288–3.946)	0.924	2.825 (0.641–12.446)	0.170
APACHE II score	1.188 (1.087–1.298)	0.000	1.268 (1.129–1.424)	0.000
Public health insurance coverage (ref. private)	0.477 (0.096–2.377)	0.366	3.233 (0.51–20.486)	0.213
N included in the model [a]	70		70	
AUC (95% CI) [d]	0.832 (0.736–0.929)	0.000	0.88 (0.796–0.964)	0.000
**Third multivariate model**	**Adjusted OR (95% CI)** [b]	**p value** [c]	**Adjusted OR (95% CI)** [b]	**p value** [c]
Male sex (ref. female)	1.82 (0.519–6.38)	0.349	3.744 (0.919–15.246)	0.065
SAPS III score	1.068 (1.027–1.111)	0.001	1.094 (1.035–1.156)	0.002
Public health insurance coverage (ref. private)	0.433 (0.101–1.844)	0.257	2.158 (0.498–9.357)	0.304
N included in the model [a]	65		65	
AUC (95% CI) [d]	0.764 (0.642–0.886)	0.001	0.8 (0.691–0.909)	0.000
**Fourth multivariate model**	**Adjusted OR (95% CI)** [b]	**p value** [c]	**Adjusted OR (95% CI)** [b]	**p value** [c]
Age (years)	1.013 (0.967–1.061)	0.587	1.018 (0.971–1.066)	0.460
Male sex (ref. female)	1.373 (0.368–5.123)	0.637	1.826 (0.483–6.905)	0.375
SOFA score	1.376 (1.143–1.655)	0.001	1.379 (1.124–1.692)	0.002
Public health insurance coverage (ref. private)	0.377 (0.063–2.256)	0.285	2.977 (0.504–17.575)	0.229
N included in the model [a]	64		64	
AUC (95% CI) [d]	0.82 (0.715–0.925)	0.000	0.812 (0.696–0.928)	0.000

[a] Number of cases (n) included in the multivariate model. [b] Odds ratios and 95% confidence intervals of the multivariate binary logistic regression model. [c] Wald test p value, results < 0.05 indicate statistical significance.
[d] Area under the receiver operating characteristic curve and 95% confidence interval. Abbreviations: ICU, intensive care unit; m-RS, modified Rankin scale score; OR, odds ratio; 95% CI, 95% confidence interval; ref., reference; AUC, area under the receiver operating characteristic curve; APACHE II, Acute Physiology and Chronic Health Evaluation II; SAPS III, Simplified Acute Physiology Score III; SOFA, Sequential Organ Failure Assessment; Hs, number of secondary injuries, resulting from the sum of the presence of hypotension, hypoxemia, hyperthermia, hypercapnia, hypocapnia, hypoglycaemia, hyponatremia, hypothermia, intracranial hypertension, and clinical evidence of herniation.

Table S17: Non-standardized Disability-adjusted life-years (DALYs), years of life lost (YLLs), and years lost due to disability (YLDs) in the overall cohort of neurocritical patients and in patients grouped by main diagnosis**.**

**Neurocritical patients**	**N**	**DALYs**	**YLLs**	**YLDs**
**Total neurocritical patients**	1194	4482.94	4420.022	62.92
Traumatic brain injury	218	1634.42	1618.800	15.62
Encephalopathy	155	709.66	703.510	6.15
Subarachnoid haemorrhage	70	559.01	557.426	1.58
Intracerebral haemorrhage	77	557.31	554.420	2.89
Ischaemic stroke	211	421.72	406.567	15.16
Central nervous system infection	25	237.13	236.589	0.54
Postoperative status of elective neurosurgery	317	195.78	180.360	15.42
Seizures	91	110.20	106.849	3.35
Spinal cord injury	19	57.54	55.500	2.04
Neuromuscular disease	11	0.17	0	0.17

Table S18: Characteristics of registered sites according to available resources

**Caracteristics of sites**	**n=36**
Located in cities with > 1 million inhabitants, n(%)	23 (63,9%)
Active hospital beds, median (IQR)	190 (136,5 - 309,75)
Adult ICU active beds, median (IQR)	34 (20 - 49)
Type of institution, n(%)	
Only public	11 (30,6%)
Only private	22 (61,1%)
Public and private	3 (8,3%)
Academic hospital, n(%)	30 (83,3%)
Availability of Cranial Tomography, n(%)	
24/7	34 (94,4%)
Elective	2 (5,6%)
Availability of Cranial Magnetic Resonance, n(%)	
24/7	19 (52,8%)
Elective	7 (19,4%)
Not available	10 (27,8%)
Availability of Cerebral Arteriography, n(%)	
24/7	22 (61,1%)
Elective	10 (27,8%)
Not available	4 (11,1%)
Hospital with surgical centre, n(%)	36 (100%)
Titled intensivist, n(%)	37 (97,2%)
Neurologist available 24/7 at the Hospital, n(%)	30 (83,3%)
Neurosurgeon available 24/7 at the Hospital, n(%)	34 (94,4%)
Professionals available in the ICU	
Dedicated Doctor, n(%)	36 (100%)
Dedicated Nurse, n(%)	36 (100%)
Dedicated Physiotherapist, n(%)	36 (100%)
Pharmacist, n(%)	32 (88,9%)
Psychologist, n(%)	31 (86,1%)
Speech therapist, n(%)	32 (88,9%)
Nutritionist, n(%)	36 (100%)
Dentist, n(%)	18 (50%)
Active care protocols in the hospital/ICU:	
Ischaemic stroke, n(%)	25 (69,4%)
Subarachnoid haemorrhage, n(%)	21 (58,3%)
Hemorrhagic stroke, n(%)	22 (61,1%)
Traumatic brain injury, n(%)	18 (50%)
External ventricular drain care, n(%)	24 (66,7%)

Deep vein thrombosis, n(%)	32 (88,9%)
Seizures, n(%)	21 (58,3%)
Bundle Ventilator-associated pneumonia, n(%)	33 (91,7%)
Bundle Urinary Infection related to urinary catheter, n(%)	33 (91,7%)
Bundle Central catheter-related infection, n(%)	32 (88,9%)
Availability EV Thrombolysis 24/7, n(%)	32 (88,9%)

Abbreviations: ICU, intensive care unit; IQR, interquartile range; n, absolute frequency; %, percentage within column.

Table S19: Comparison of the characteristics of patients with a primary diagnosis of encephalopathy, stratified by etiologies.

**Variables**	septic encephalopathy (n=64)	brain structural damage (n=26)	metabolic encephalopathy (n=21)	hypoxic-ischaemic encephalopathy (n=19)	drug-induced encephalopathy (n=13)	other etiologies (n=12)
**Age (years),** mean ± SD	74.2 ± 15.9	53 ± 17.6	68.9 ± 19.3	61 ± 19.1	44.4 ± 21.9	72.4 ± 17.1
**Male Sex**, n (%)	33 (51.6)	10 (38.5)	5 (23.8)	16 (84.2)	4 (30.8)	4 (33.3)
**GCS**, Median (IQR)	12 (10 - 14)	14 (10 - 15)	13 (9 - 14)	12 (3 - 14)	6 (4 - 13)	14 (12.5 - 14.5)
**APACHE II at ICU admission**, Median (IQR)	17 (13 - 25)	9 (7 - 11)	20 (11 - 26)	30 (20 - 35)	12 (4 - 22)	11 (8.5 - 17.5)
**SAPS III at ICU admission**[a], Median (IQR)	60 (52 - 71)	49 (43 - 58)	55 (50 - 62)	67 (49 - 86)	49.5 (38 - 66.5)	52 (46 - 60)
**SOFA at ICU admission**, Median (IQR)	6 (3 - 8)	2 (1 - 4)	5 (3 - 9)	9 (7 - 12)	4 (1 - 7)	2 (0.5 - 2.5)
**Number of Hs at ICU admission**, n (%)						
Zero	17 (26.6)	13 (50)	4 (19)	3 (15.8)	5 (38.5)	5 (41.7)
One	14 (21.9)	3 (11.5)	7 (33.3)	3 (15.8)	6 (46.2)	6 (50)
Two	16 (25)	6 (23.1)	6 (28.6)	2 (10.5)	1 (7.7)	1 (8.3)
Three or more	17 (26.6)	4 (15.4)	4 (19)	11 (57.9)	1 (7.7)	0 (0)
**Length of ICU stay until 30th day,** Median (IQR)	5.5 (3 - 12)	8 (5 - 15)	3 (2 - 17)	6 (4 - 12)	3.5 (1.5 - 15.5)	3 (2 - 4)
**30-day mortality**, n (%)	20 (31.3)	5 (19.2)	3 (14.3)	11 (57.9)	1 (7.7)	1 (8.3)
**m-RS on ICU outcome or until 30th day**, n (%)						
0	6 (9.4)	2 (7.7)	4 (19)	0 (0)	4 (30.8)	2 (16.7)
1	6 (9.4)	2 (7.7)	3 (14.3)	0 (0)	3 (23.1)	2 (16.7)
2	4 (6.3)	7 (26.9)	1 (4.8)	0 (0)	1 (7.7)	3 (25)
3	9 (14.1)	1 (3.8)	2 (9.5)	2 (10.5)	1 (7.7)	1 (8.3)
4	10 (15.6)	6 (23.1)	2 (9.5)	3 (15.8)	2 (15.4)	1 (8.3)
5	9 (14.1)	3 (11.5)	6 (28.6)	3 (15.8)	1 (7.7)	2 (16.7)
6	20 (31.3)	5 (19.2)	3 (14.3)	11 (57.9)	1 (7.7)	1 (8.3)
**Unfavourable outcome (m-RS score 4, 5, or 6)**, n (%)	39 (60.9)	14 (53.8)	11 (52.4)	17 (89.5)	4 (30.8)	4 (33.3)

Abbreviations: APACHE II, Acute Physiology and Chronic Health Evaluation; ENC, encephalopathy; GCS, Glasgow Coma Scale; ICH, intracerebral haemorrhage; IQR, interquartile range; IS, ischaemic stroke; m-RS, modified Rankin Scale; n, absolute frequency; NMD, neuromuscular disease; NPO, postoperative care of elective neurosurgery; SAH, subarachnoid haemorrhage; SAPS III, Simplified Acute Physiology Score III; SCI, spinal cord injury; SD, standard deviation; SNI, central nervous system infection; SOFA, Sequential Organ Failure Assessment; TBI, traumatic brain injury; %, percentage within column.

[a] Missing data on SAPS III: 1 on drug-induced encephalopathy.
